# Supplementary material for: Telomere-to-telomere genome assembly and a pangenome for the rat
Source: Cell Genom. 2026 Aug 6;6(8):101281. doi: 10.1016/j.xgen.2026.101281 (PMC13477028; doi:10.1016/j.xgen.2026.101281)
Supplement: Supplementary file 1 [file mmc1.pdf]

Cell Genomics, Volume *6*

Supplemental information

Telomere-to-telomere genome assembly

and a pangenome for the rat

Kai Li, Julia L. Ciosek, Sergey Koren, Adam M. Phillippy, Yaming Zhu, William A. Lauer, Shelise Y. Brooks, Gerard G. Bouffard, Brandon D. Pickett, Beth L. Dumont, Melissa L. Smith, Theodore S. Kalbfleisch, and Peter A. Doris

Supplemental Figure and Table legends

Fig. S1. An illustration of the various sequencing and bioinformatic processes used to create the assembly, **related to Star Methods –** Method Details: sequencing and assembly

Fig. S2. K-mer analysis by Meryl/Merqury to validate haploid genome, **related to Star Methods –** Method Details: sequencing and assembly

Fig. S3. ModDotPlot self-similarity analysis (color encoded with a calibrated spectrum included) to identify potential centromeric regions, **related to Star Methods –** Method Details: sequencing and assembly

Fig. S4. IGV session for a duplicated region on chr19 showing unshaded MQ=0 short reads that impede variant analysis and positionally accurate genotyping in this region, **related to Star Methods**: Scripts for identifying duplicated regions resistant to short read evaluation

Fig. S5. Non-allelic homologous recombination (NAHR) evaluation using NAHRwhals software in 4 genomic regions in which gene duplication events were observed, **related to Tables 3 and 4**, identification of novel genes

Table S1. Chromosome QV scores estimated by Merqury using the Illumina, HiFi and ONT reads from which the assembly was created, **related to Table 1B,** overall assembly statistics for the SHRSP T2T assembly compared to the prior SHRSP HiFi assembly

Table S2. Telomere lengths in the assembly, **related to Table 1B,** overall assembly statistics for the SHRSP T2T assembly compared to the prior SHRSP HiFi assembly

Table S3. Rat and mouse genome location of human PAR1 genes associated with chr X and Y, **related to Figure 3A,** PAR1 region of X and Y shows sequence similarity in humans, but not in rats

Table S4. Duplicated regions in the SHRSP T2T genome in which short read mapping results in extended regions of non-zero mapped reads that are >100kb, **related to Star Methods** : Scripts for identifying duplicated regions resistant to short read evaluation

Table S5. Duplicated regions are fewer in the previous assembly (GCA_021556685.1) constructed without ONT-UL data, **related to Star Methods** : Scripts for identifying duplicated regions resistant to short read evaluation

Fig. S1

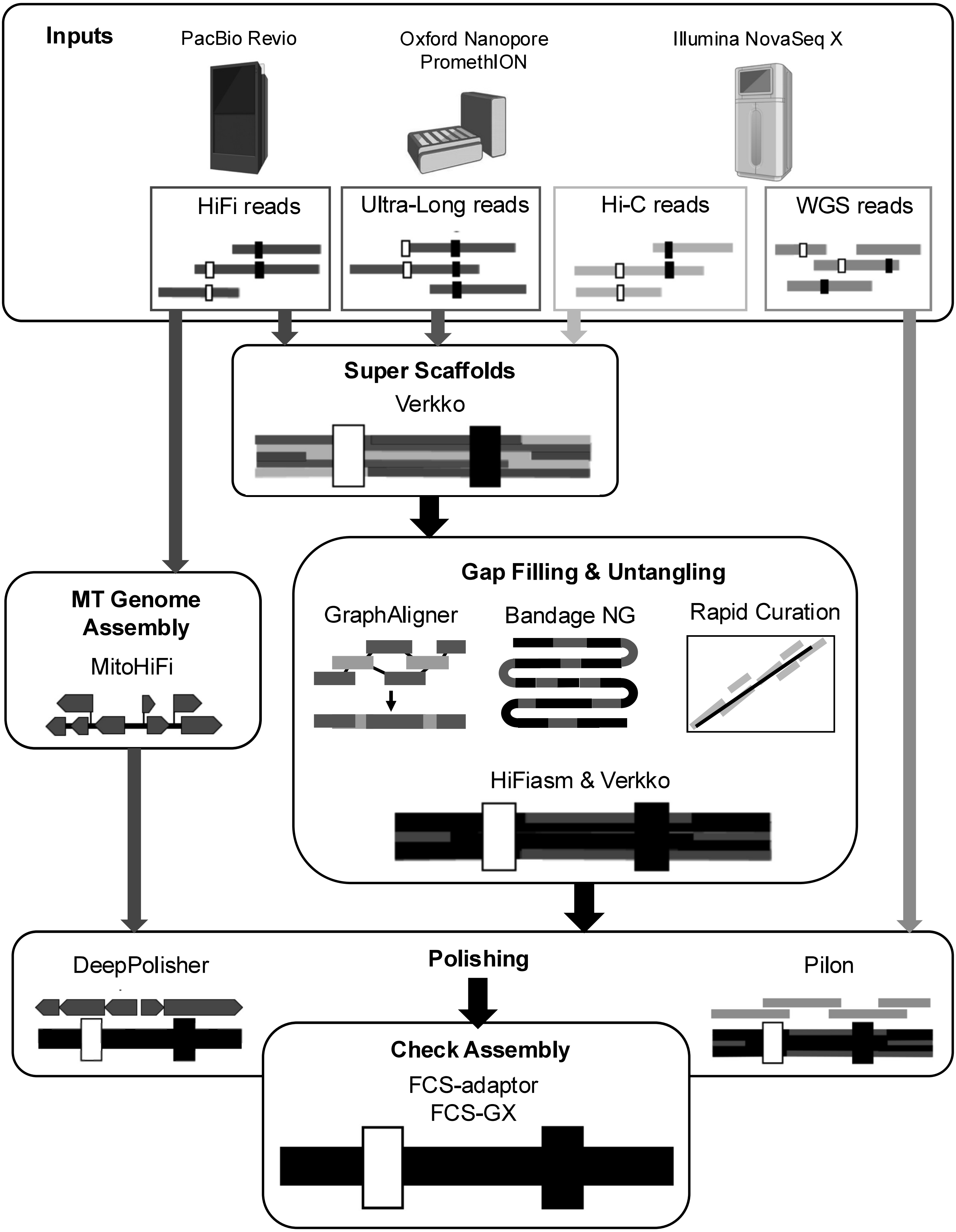

Inputs
PacBio Revio
Oxford Nanopore PromethION
Illumina NovaSeq X
HiFi reads
Ultra-Long reads
Hi-C reads
WGS reads
Super Scaffolds
Verkko
MT Genome Assembly
MitoHiFi
Gap Filling & Untangling
GraphAligner
Bandage NG
Rapid Curation
HiFiasm & Verkko
DeepPolisher
Polishing
Pilon
Check Assembly
FCS-adaptor
FCS-GX

Fig. S2 K−mer Frequency Distribution

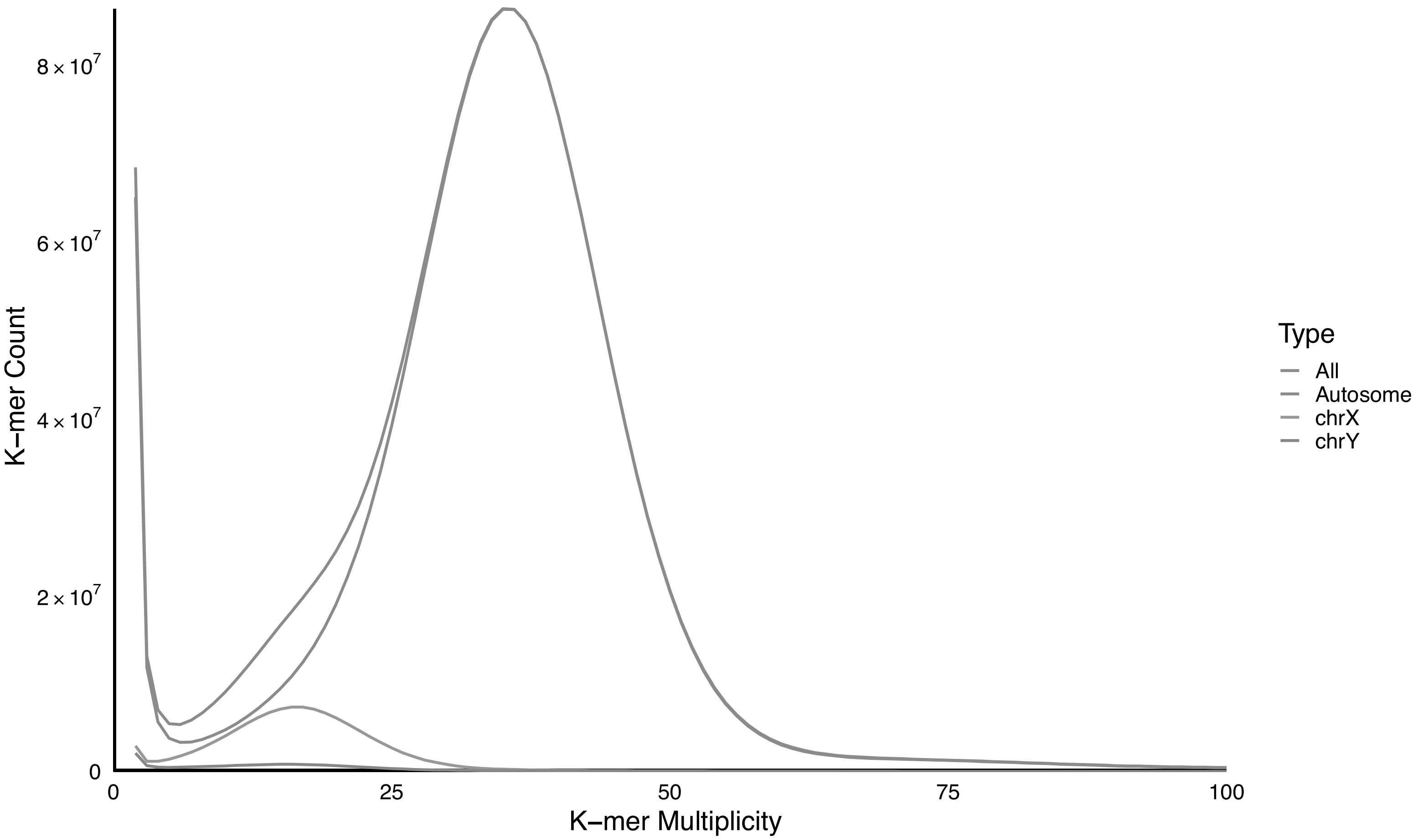

Fig. S3

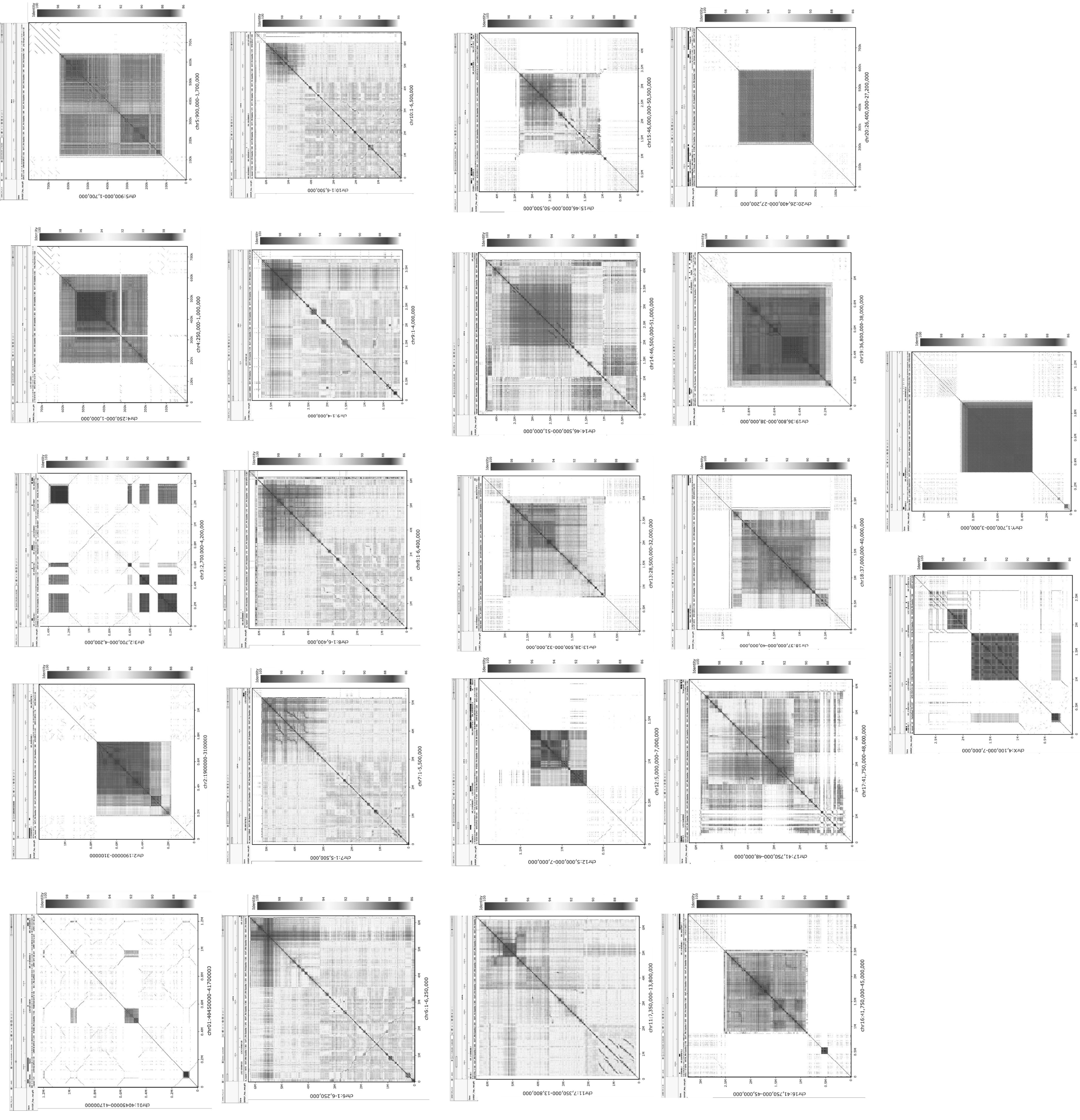

Identity
chr5:900,000-1,700,000
chr10:1-6,500,000
chr15:46,000,000-50,500,000
chr20:26,400,000-27,200,000
chr4:250,000-1,000,000
chr9:1-4,000,000
chr14:46,500,000-51,000,000
chr19:36,800,000-38,000,000
chrY:1,700,000-3,000,000
chr3:2,700,000-4,200,000
chr8:1-6,400,000
chr13:28,500,000-32,000,000
chr18:37,000,000-40,000,000
chrX:4,100,000-7,000,000
chr2:1900000-3100000
chr7:1-5,500,000
chr12:5,000,000-7,000,000
chr17:41,750,000-48,000,000
chr01:40450000-41700000
chr6:1-6,250,000
chr11:7,350,000-13,800,000
chr16:41,750,000-45,000,000

Fig. S4

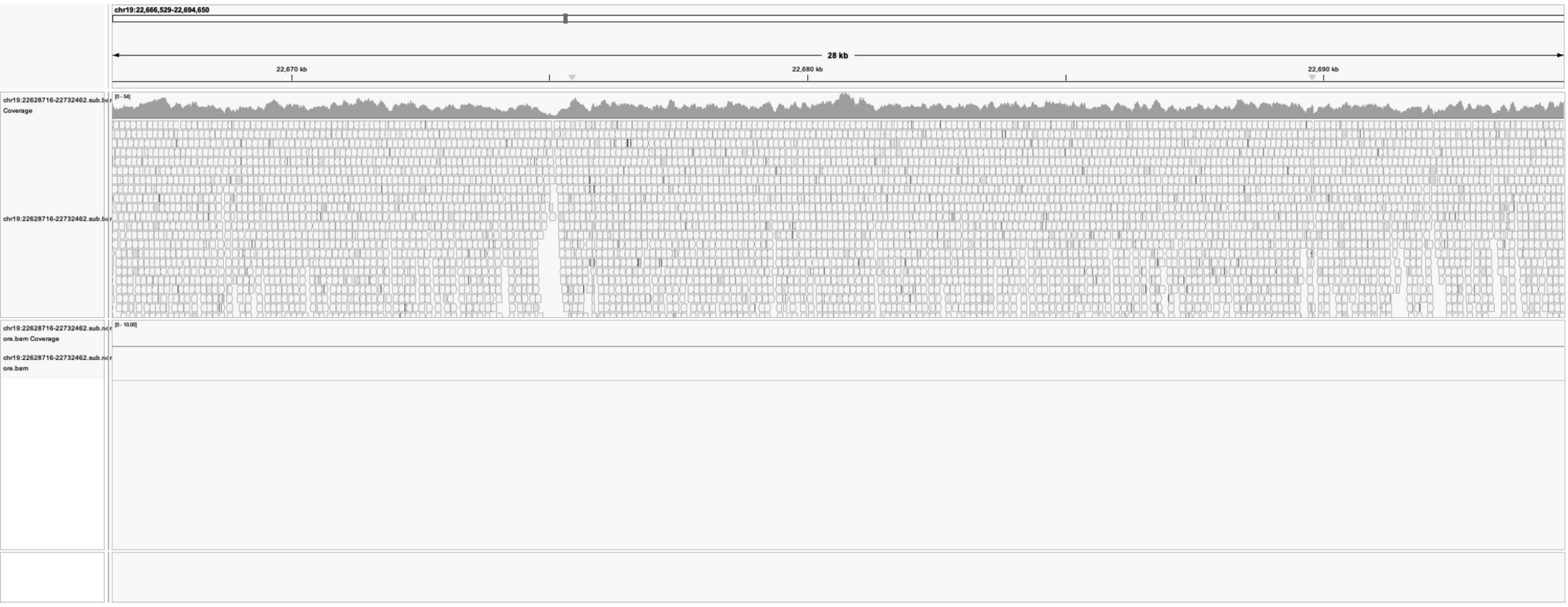
chr19:22,666,529-22,694,650
28 kb
22,670 kb
22,680 kb
22,690 kb
chr19:22628716-22732462.sub.ba
Coverage
[0 - 54]
chr19:22628716-22732462.sub.ba
chr19:22628716-22732462.sub.nc
ore.bam Coverage
[0 - 10.00]
chr19:22628716-22732462.sub.nc
ore.bam

Fig. S5

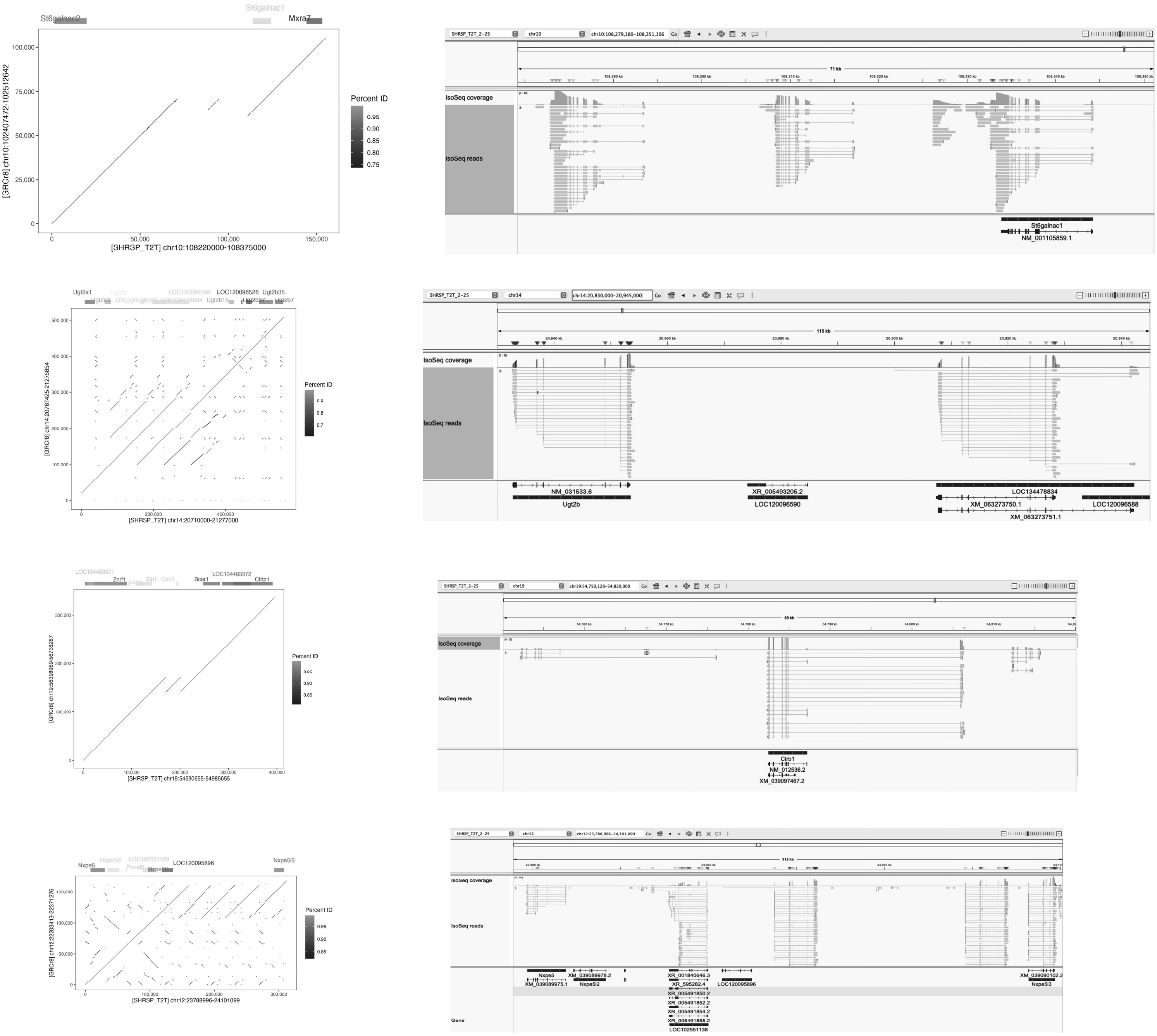

St6galnac2
St6galnac1
Mxra7
[GRCr8] chr10:102407472-102512642
[SHRSP_T2T] chr10:108220000-108375000
Percent ID
SHRSP_T2T_2-25
chr10
chr10:108,279,180-108,351,106
71 kb
IsoSeq coverage
IsoSeq reads
St6galnac1
NM_001105859.1
Ugt2a1
LOC120096526
Ugt2b35
[GRCr8] chr14:20767425-21275854
[SHRSP_T2T] chr14:20710000-21277000
chr14
chr14:20,830,000-20,945,000
115 kb
NM_031533.6
Ugt2b
XR_005493205.2
LOC120096590
LOC134478834
XM_063273750.1
XM_063273751.1
LOC120096588
LOC134483371
Znrf1
Bcar1
LOC134483372
Cfdp1
[GRCr8] chr19:56399959-56733287
[SHRSP_T2T] chr19:54590655-54985655
chr19
chr19:54,750,128-54,820,000
69 kb
Ctrb1
NM_012536.2
XM_039097467.2
Nxpe5
LOC120095896
Nxpe5l3
[GRCr8] chr12:22203413-22371276
[SHRSP_T2T] chr12:23788996-24101099
chr12
chr12:23,788,996-24,101,099
312 kb
Nxpe5
XM_039089975.1
XM_039089978.2
Nxpe5l2
XR_001840646.3
XR_595282.4
XR_005491850.2
XR_005491852.2
XR_005491854.2
XR_005491866.2
LOC102551138
XM_039090102.2
Nxpe5l3
Gene

Table S1

QV of assembled genome

Chromosome	Uniq kmer	Total kmer	QV	Error rate
1	1225	273974597	66.7	2.13E-07
2	1191	251298673	66.5	2.26E-07
3	198	169841121	72.6	5.55E-08
4	487	184837503	69.0	1.25E-07
5	716	173011393	67.1	1.97E-07
6	133	146793962	73.7	4.31E-08
7	113	144522209	74.3	3.72E-08
8	107	131669335	74.1	3.87E-08
9	182	129549271	71.7	6.69E-08
10	84	113562547	74.5	3.52E-08
11	114	99913065	72.6	5.43E-08
12	185	53687143	67.8	1.64E-07
13	2901	109399675	59.0	1.26E-06
14	38	109260667	77.8	1.66E-08
15	33	107424533	78.3	1.46E-08
16	427	92830094	66.6	2.19E-07
17	65	94106387	74.8	3.29E-08
18	1245	85849053	61.6	6.91E-07
19	16	72640477	79.8	1.05E-08
20	271	57885805	66.5	2.23E-07
X	8184	162181918	56.2	2.40E-06
Y	1138	77330154	61.5	7.01E-07
Assembly QV=	65.0	Autosomes QV=	67.5	

Table S2

Telomeric repeat lengths by chromosome/arm in SHRSP T2T assembly. The average length was calculated using TeloBP (see Methods) and the maximum length is the length of the longest telomeric repeat read counted from the telomeric boundary identified by TeloBP.

	**Telomere length (bases)**			**Telomere length (bases)**	
	Average	Max		Average	Max
chr01_p-arm	31900	103106	chr01_q-arm	29651	74025
chr02_p-arm	26291	54939	chr02_q-arm	30758	76861
chr03_p-arm	10112	84021	chr03_q-arm	29189	63066
chr04_p-arm	29721	80928	chr04_q-arm	30582	72958
chr05_p-arm	38509	77090	chr05_q-arm	24833	147301
chr06_p-arm	34734	70787	chr06_q-arm	25271	72188
chr07_p-arm	37333	74893	chr07_q-arm	35225	70606
chr08_p-arm	28734	147301	chr08_q-arm	43705	128050
chr09_p-arm	33070	62410	chr09_q-arm	34782	147301
chr10_p-arm	37289	125297	chr10_q-arm	26701	80664
chr11_p-arm	32829	88879	chr11_q-arm	31954	78136
chr12_p-arm	33951	75358	chr12_q-arm	33576	83134
chr13_p-arm	37409	90113	chr13_q-arm	27669	74560
chr14_p-arm	32854	70048	chr14_q-arm	30669	63963
chr15_p-arm	31232	82048	chr15_q-arm	33368	85294
chr16_p-arm	48196	128050	chr16_q-arm	27686	68917
chr17_p-arm	36109	73528	chr17_q-arm	34513	103215
chr18_p-arm	30499	68127	chr18_q-arm	22984	51146
chr19_p-arm	41015	116710	chr19_q-arm	32817	77650
chr20_p-arm	30071	78195	chr20_q-arm	40097	123928
chrX_p-arm	39629	90148	chrX_q-arm	29676	62908
chrY_p-arm	33057	57004	chrY_q-arm	24481	65704

Table S3. Genomic location of genes associated with chr X and Y human PAR1 in rat and mouse genome assemblies. The first panel shows gene in the human PAR1 from the telomere to the region of Xg. The second panel shows other genes conserved in the adjacent human chrX and there location in mouse and rat assemblies.

Gene	Hsa RefSeq	GRCh38	Mmu RefSeq	GRCm39 position	Rn RefSeq	GRCr8 position	Rn RefSeq	SHRSP T2T position
PLCXD1	NM_018390	chrX:281381-303356	NM_207279.3	chr5:110247661-110253819	XM_006249594.4	chr12:52,238,611-52,241,876	XM_006249594.4	chr12:53,611,726-53,616,155
		chrY:281381-303356						
GTPBP6	NM_012227	chrX:304759-318796	NM_145147.5	chr5:110251843-11025606	NM_001135840.1	chr12:52,234,612-52,238,574	NM_001135840.1	chr12:53,607,932-53,612,854
		chrY:304759-318796						
PPP2R3B	NM_013239	chrX:333933-386907		absent	NM_001139492.1	chr14:103,483-110,740	NM_001139492.1	chr14:100,844-109,075
		chrY:333933-386907						
SHOX	NM_001191546	chrX:630898-644636	NM_001302357.	chr3:66879056-66889104	XM_001077477.5		XM_001077477.5	
		chrY:630898-644636				chrX:121,553,770-121,560,78		chrX:125,394,483-125,402,485
CRLF2	NM_022148	chrX:1190490-1212649	NM_001310694.	chr5:109702575-109706859	NM_134465.2	chr14:118,930-123,637	NM_134465.2	chr14:116,268-121,942
		chrY:1190490-1212649						
CSF2RA	NM_001161531	chrX:11268814-1305941	NM_009970.2	chr19:61212840-61216856	NM_001037660.1	chr14:113,073-118,247	NM_001037660.1	chr14:110,559-116,453
		chrY:1268814-1305941						
IL3RA	NM_002183	chrX:1336785-1382689	NM_008369.2	chr14:8114270-8123357	NM_139260.3	chr12:21,431,846-21,437,547	NM_139260.3	chr12:23,010,514-23,016,9
		chrY:1336785-1382689						
SLC25A6	NM_214418	chrX:1386596-1392042		absent	XM_039097301.1	chr18:70,846,358-70,847,597	XM_039097301.1	chr18:70,601,165-70,603,404
		chrY:1386157-1392042						
ASMTL	NM_004192	chrX:1403139-1452909		absent	NM_001105915.2	chr12:21,429,098-21,431,849	NM_001105915.2	chr12:23,007,850-23,011,513
		chrY:1403139-1452909						
P2RY8	NM_178129	chrX:1462581-1537185		absent		absent		absent
		chrY:1462581-1537185						
AKAP17A	NM_005088	chrX:1591604-1602520	NM_001418372.	chrX:169098201-169105547	NM_001127246.2	chr12:21,424,191-21,428,933	NM_001127246.2	chr12:23,002,870-23,007,915
		chrY:1591604-1602520						
ASMT	NM_001171038.2	chrX:1615059-1643081	NM_001308488.	chrX:169106356-169111844	NM_144759.2	chr12:21,418,488-21,423,338	NM_144759.2	chr12:22,997,036-23,003,004
		chrY:1615059-1643081						
DHRSX	NM_145177	chrX:2219506-2500976	NM_001033326.	chr4:156390377-156410432	XM_063271142.1	chr12:21,413,533-21,418,321	XM_063271142.1	chr12:22,992,105-22,997,868
		chrY:2219506-2500976						
ZBED1	NM_004729	chrX:2486435-2500976		absent		absent		absent
		chrY:2486435-2500976						
CD99	NM_002414	chrX:2691295-2741309	NM_138309.3	chrX:70463666-70536455	XM_063279501.1	chr20:56,000,484-56,005,135	XM_063279501.1	chr20:57,753,061-57,758,710
		chrY:2691295-2741309						
XG	NM_001141919	chrX:2752040-2816500		absent		absent		absent
		chrY:2752052-2752360						

Gene	Hsa RefSeq	GRCh38 Position	Mmu RefSeq	GRCm39 position	Rn RefSeq	GRCr8 position	Rn RefSeq	SHRSP T2T position
Gyg2	NM_001184704.2	chrX:2,828,430-2,883,318		absent		absent		absent
Arsd	NM_009589.5	chrX:2,903,472-2,929,839		absent		absent		absent
Arsl	NM_000047.3	chrX:2,934,309-2,968,745		absent		absent		absent
Arsh	NM_001011719.2	chrX:3,006,046-3,034,611		absent		absent		absent
Arsf	NM_001201538.2	chrX:3,039,734-3,113,227		absent		absent		absent
Mxra5	NM_015419.4	chrX:3,308,065-3,347,152		absent		absent		absent
Prkx	NM_005044.5	chrX:3,603,840-3,714,149	NM_001411694.1	chrX:76,804,078-76,840,045	NM_001033963.2	chrX:45,701,247-45,745,710	NM_001033963.2	chrX:49,612,943-49,657,417
Nlgn4	NM_001441322.1	chrX:5,889,542-6,229,367		absent		absent		absent
Vcx3a	NM_016379.4	chrX:6,533,118-6,535,618		absent		absent		absent
Pudp	XR_007068202.1	chrX:6,705,338-7,148,653	NM_026108.3	chr18:50,700,227-50,702,270	NM_001419508.1	chr18:46,064,367-46,067,747	NM_001419508.1	chr18:45,869,392-45,872,812
Sts	NM_001320752.2	chrX:7,146,790-7,355,141	NM_009293.1	chrX:168,855,832-169,015,537	NM_012661.1	chrX:46,102,024-46,111,368	NM_012661.1	chrX:50,028,597-50,037,940
Vcx	NM_013452.3	chrX:7,841,762-7,844,643		absent		absent		absent
Pnpla4	NM_004650.3	chrX:7,897,747-7,928,224		absent	NM_001401182.1	chrX:46,199,851-46,206,017	NM_001401182.1	chrX:50,131,310-50,137,481
Anos1	NM_001161531	chrX:8,528,374-8,732,637		absent	XM_002727786.4	chr10:69,058,951-69,060,798	XM_002727786.4	chr10:74,472,991-74,474,838
Fam9a	NM_174951.3	chrX:8,790,295-8,801,883		absent		absent		absent
Fam9b	NM_205849.3	chrX:9,023,732-9,034,627		absent	NM_001408910.1	chrX:138,541,821-138,555,147	NM_001408910.1	chrX:142,317,404-142,330,730
Tbl1x	NM_05647.4	chrX:9,462,795-9,720,240	NM_001426017.1	chrX:76,552,553-76,707,091	XM_039099666.2	chrX:45,452,483-45,610,032	XM_039099666.2	chrX:49,352,899-49,510,378
Gpr143	NM_001440781.1	chrX:9,724,846-9,779,102	NM_010951.3	chrX:151,564,417-151,592,142	NM_00116958.1	chrX:25,498,101-25,523,908	NM_00116958.1	chrX:31,429,925-31,922,719
Shroom2	NM_001649.4	chrX:9,785,929-9,949,943	NM_001290687.2	chrX:151,392,005-151,552,982	NM_001047893.3	chrX:25,307,663-25,479,924	NM_001047893.3	chrX:29,104,315-29,276,644
Wwc3	NM_0015691.5	chrX:10,014,754-10,144,974		absent	NM_001427611.1	chrX:27,145,518-27,263,946	NM_001427611.1	chrX:30,946,849-31,063,942
Clcn4	NM_001256994.2	chrX:10,156,475-10,238,160	NM_001357102.2	chr7:7,284,808-7,304,337	NM_001135840.1	chrX:27,290,269-27,357,467	NM_001135840.1	chrX:31,090,269-31,157,494
Mid1	NM_033290.4	chrX:10,444,810-10,834,183	NM_001425885.1	chrX:168,467,695-168,774,192	NM_022927.1	chrX:27,677,748-28,053,549	NM_022927.1	chrX:31,485,015-31,867,629

Table S4

Regions in this assembly that are effectively invisible for the purposes of variant analysis in genome wide association studies. These regions were identified by looking for distances >100kb between adjacent variants in a cohort VCF file called against the SHRSP T2T assembly using Illumina read data of 8 distinct inbred rat strains. The table lists regions in the SHRSP T2T genome that are >100kb and are invariant across strains.

Region	Total_Mapped Reads	Nonzero_Reads	Region_Length	Nonzero_Ratio	Depth_Include Zero_Reads
chr01:65528461-65828410	143,022	264	299,950	0.185%	46.7054
chr06:5375127-5547572	140,259	1137	172,446	0.811%	79.0851
chr08:0-101533	60,322	850	101,534	1.409%	21.4942
chr16:22944986-23138054	98,007	415	193,069	0.423%	49.6452
chr16:23152915-23333782	91,162	107	180,868	0.117%	49.3319
chr19:13788478-13988717	57,192	215	200,240	0.376%	27.914
chr19:13988718-14963881	246,526	27	975,164	0.011%	24.7821
chr19:14964518-15222277	64,371	2	257,760	0.003%	24.4403
chr19:15222278-15795262	145,384	65	572,985	0.045%	24.8215
chr19:15796756-16094218	77,606	2	297,463	0.003%	25.5164
chr19:16094789-16229615	34,119	0	134,827	0.000%	24.7506
chr19:16370485-16629041	66,845	2	258,557	0.003%	25.3348
chr19:16630401-17320124	164,482	37	689,724	0.022%	23.3737
chr19:17446618-18280396	202,076	4	833,779	0.002%	23.7504
chr19:18281443-19281592	247,717	42	1,000,150	0.017%	24.296
chr19:19282649-19916613	154,570	2	633,965	0.001%	23.9308
chr19:19916930-20408635	123,754	8	491,706	0.006%	24.6501
chr19:20599688-20708177	27,188	0	108,490	0.000%	24.4703
chr19:20708509-21649032	245,579	17	940,524	0.007%	25.5513
chr19:21904580-22083155	45,437	36	178,576	0.079%	24.9547
chr19:22083756-22341447	65,268	260	257,692	0.398%	24.7863
chr19:22356391-22592830	60,612	5	236,440	0.008%	25.1152
chr19:22628716-22732462	26,095	3	103,747	0.011%	24.6093
chr19:23005894-23193471	47,791	1	187,578	0.002%	24.9436
chr19:23511545-23671886	40,937	6	160,342	0.015%	25.0209

chr19:23672912-23798222	31,495	4	125,311	0.013%	24.5741
chr19:23801399-24277146	109,628	4	475,748	0.004%	22.6075
chr19:24409177-24530134	30,649	2	120,958	0.007%	24.8296
chr19:24604743-24711202	27,104	2	106,460	0.007%	24.9446
chr19:24770895-25326162	139,490	8	555,268	0.006%	24.583
chr19:25459227-25614553	39,514	6	155,327	0.015%	24.8812
chr19:25672011-25792543	34,003	26	120,533	0.076%	27.3368
chr19:25839357-25972814	33,038	6	133,458	0.018%	24.2523
chr19:25973876-26654632	173,897	140	680,757	0.081%	25.0414
chr19:26765190-27047607	96,337	10	282,418	0.010%	33.4786
chr19:27047639-27150332	36,398	116	102,694	0.319%	34.7554
chr19:27151213-27383024	78,759	0	231,812	0.000%	33.3559
chr19:27384256-27485273	33,380	7	101,018	0.021%	32.4317
chr19:27598238-27759195	57,443	0	160,958	0.000%	35.0311
chr19:27770887-27937813	58,514	3	166,927	0.005%	34.3925
chr20:39944072-40079376	70,996	392	135,305	0.552%	51.0319
chr20:40079812-40229066	77,718	528	149,255	0.679%	50.7071
chr20:57765266-57865713	105,542	8923	100,448	8.454%	33.098
chrX:0-197365	44,399	294	197,366	0.662%	15.574
chrX:198171-381930	16,859	50	183,760	0.297%	8.86722
chrX:383202-576381	18,286	38	193,180	0.208%	9.09314
chrX:586322-752104	23,877	56	165,783	0.235%	13.8111
chrX:795043-1121140	34,027	13	326,098	0.038%	10.0386
chrX:1413954-1617324	21,861	0	203,371	0.000%	10.3957
chrX:1617597-1726332	17,863	6	108,736	0.034%	15.7573
chrX:1727952-1885204	28,328	7	157,253	0.025%	17.5036
chrX:1971370-2179354	15,854	0	207,985	0.000%	7.38946
chrX:2192190-2395894	30,045	21	203,705	0.070%	14.1639
chrX:2545378-2733028	24,881	32	187,651	0.129%	12.7863
chrX:2733530-3149204	59,864	120	415,675	0.200%	13.9084
chrX:3149449-3267966	14,585	1	118,518	0.007%	11.8785
chrX:3268380-3744500	55,355	96	476,121	0.173%	11.1987

chrY:2016132-2127445	20,257	194	111,314	0.958%	17.9811
chrY:2267198-2514583	46,212	897	247,386	1.941%	18.4785
chrY:76844035-77331694	72,831	191	487,660	0.262%	11.363
		Sum of regions	17,363,793		

Table S5

Regions in this assembly >100kb between adjacent variants that are effectively invisible for the purposes of variant analysis in genome wide association studies are reduced when the cohort variants are called against the HiFi assembly of SHRSP (GCA_021556685.1). The table lists regions in the SHRSP HiFi genome that are invariant across strains. Significantly fewer invariant regions are called than in Table S4 reflecting the ability of the T2T assembly to more faithfully represent duplication events in the genome.

**Region**	**Total Mapped Reads**	**Nonzero Reads**	**Region Length**	**Nonzero Ratio**	**Depth_Include Zero_Reads**
chr1:1928585-2035493	21513	27	106909	0.126%	19.5536
chr1:2893349-3005595	24006	55	112247	0.229%	20.7701
chr2:3373804-3507174	39866	138	133371	0.346%	29.0775
chr3:182367925-182472286	36256	380	104362	1.048%	24.8097
chr3:182502840-182605066	20399	89	102227	0.436%	19.2104
chr4:186772362-186908652	24619	58	136291	0.236%	17.4876
chr4:186909387-187063658	22874	74	154272	0.324%	14.3632
chr6:142019143-142131637	45392	811	112495	1.787%	39.3922
chr7:54149-170508	23738	14	116360	0.059%	19.7701
chr7:229524-329888	21315	16	100365	0.075%	20.5728
chr7:2485313-2701255	615	57	215943	9.268%	0.264418
chr9:20230597-20341807	13083	433	111211	3.310%	11.4481
chr10:10667-167918	27656	106	157252	0.383%	16.9395
chr15:596915-706760	24471	18	109846	0.074%	21.5526
chr15:1105811-1222413	24712	54	116603	0.219%	20.465
chr15:7112818-7305166	4255	0	192349	0.000%	2.1544
chr16:960491-1082621	24687	55	122131	0.223%	19.5232
chr17:0-221720	39352	121	221721	0.307%	17.2219
chr17:376911-580750	40955	549	203840	1.340%	19.3812
chr19:14508370-14637801	10864	3	129432	0.028%	8.18922
chr20:56063334-56165450	19040	52	102117	0.273%	18.0339
chrY:2064322-2355205	54357	6864	290884	12.628%	17.6654
chrY:34604250-34776359	1456	0	172110	0.000%	0.820939
		Sum of regions	3324338		
