# Supplementary material for: Telomere-to-telomere genome assembly and a pangenome for the rat
Source: Cell Genom. 2026 Aug 6;6(8):101281. doi: 10.1016/j.xgen.2026.101281 (PMC13477028; doi:10.1016/j.xgen.2026.101281)
Supplement: Supplementary file 2 [file mmc2.pdf]

Methods S1

Rubric for novel gene ID from BRAKER and SHRSP 19 tissue HQ IsoSeq

1. The novel gene must not appear in the liftoff annotation of SHRSP from GRCr8
2. The novel gene must have a transcript in the 19 tissue SHRSP IsoSeq HQ collection
3. The novel gene must not overlap an existing annotated gene, unless more than 2 new exons are indicated
4. The novel gene must have two or more exons that overlap the BRAKER annotated exons
5. The aligned IsoSeq transcript must be a primary alignment
