# Supplementary material for: Telomere-to-telomere genome assembly and a pangenome for the rat
Source: Cell Genom. 2026 Aug 6;6(8):101281. doi: 10.1016/j.xgen.2026.101281 (PMC13477028; doi:10.1016/j.xgen.2026.101281)
Supplement: Supplementary file 3 [file mmc4.pdf]

Cell Genomics

Article

Telomere-to-telomere genome assembly and a pangenome for the rat

Graphical abstract

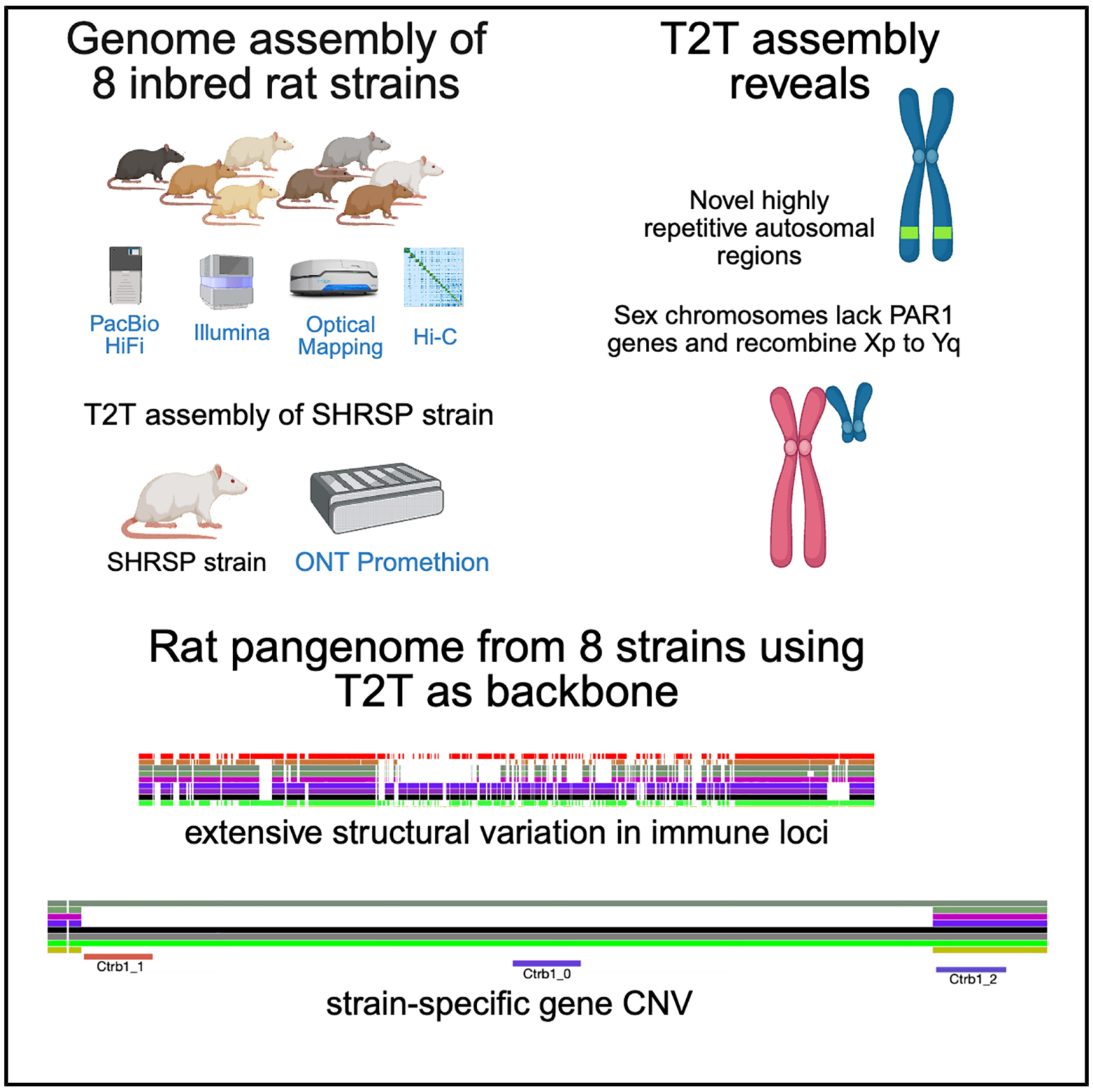

Highlights

- A telomere-to-telomere (T2T) assembly of a rat genome
- Novel genes were identified using gene prediction software and long-read RNA-seq
- Sex chromosomes indicated loss of PAR genes and meiosis between Xp and Yq
- Demonstrate rat strain genomic relationships by creating an 8-strain pangenome

Authors

Kai Li, Julia L. Ciosek, Sergey Koren, ..., Melissa L. Smith, Theodore S. Kalbfleisch, Peter A. Doris

Correspondence

peter.a.doris@uth.tmc.edu

In brief

Rat strains have been used in a wide range of genetic studies. Here, the rat genome is advanced through the creation of a T2T assembly that provides novel insights into genome content and function. The pangenome permits inter-strain genomic analysis.

Li et al., 2026, Cell Genomics *6*, 101281
August 12, 2026 © 2026 The Authors. Published by Elsevier Inc.
https://doi.org/10.1016/j.xgen.2026.101281

CellPress

Cell Genomics

CellPress
OPEN ACCESS

Article

Telomere-to-telomere genome assembly and a pangenome for the rat

Kai Li,[1] Julia L. Ciosek,[1] Sergey Koren,[2] Adam M. Phillippy,[2] Yaming Zhu,[3] William A. Lauer,[4] Shelise Y. Brooks,[5] Gerard G. Bouffard,[5] Brandon D. Pickett,[2] Beth L. Dumont,[6] Melissa L. Smith,[4] Theodore S. Kalbfleisch,[1] and Peter A. Doris[3,7,*]

[1]Department of Veterinary Science, Martin-Gatton College of Agriculture, Food, and Environment, University of Kentucky, Lexington, KY, USA
[2]Genome Informatics Section, Center for Genomics and Data Science Research, National Human Genome Research Institute, National Institutes of Health, Bethesda, MD, USA
[3]Center for Human Genetics, Brown Foundation Institute of Molecular Medicine, McGovern Medical School, University of Texas Health Science Center, Houston, TX, USA
[4]Department Biochemistry and Molecular Biology, University of Louisville School of Medicine, Louisville, KY, USA
[5]NIH Intramural Sequencing Center, National Human Genome Research Institute, National Institutes of Health, Bethesda, MD, USA
[6]The Jackson Laboratory, 600 Main Street, Bar Harbor, ME, USA
[7]Lead contact
*Correspondence: peter.a.doris@uth.tmc.edu
https://doi.org/10.1016/j.xgen.2026.101281

SUMMARY

We report a complete rodent telomere-to-telomere genome assembly from the brown rat, *Rattus norvegicus*. Annotation was enriched with multi-tissue long-read RNA sequencing and uncovered numerous novel genes. Assembly of both sex chromosomes reveals the absence of gene coding in the presumed pseudo-autosomal regions and the presence of centromeric satellite repeats on distal chromosome Y (chrY). We provide evidence of meiotic conjunction between Xp and Yq. The genome assembly reveals several expanded autosomal regions enriched for testis-expressed genes. Finally, we have generated a pangenome from recent high-quality assemblies of 8 distinct inbred rat strain genomes. This allows the strain-specific distribution of structural variation to be examined. Non-allelic homologous recombination has produced multiple copies of several genes with evidence of transcription from duplicated copies.

INTRODUCTION

Genome assembly of the rat, *Rattus norvegicus*, has been lower in quality compared to human and mouse genomes.[1] However, recently we have generated 8 reference-quality genome assemblies from 8 distinct inbred rat strains. These strains provide models of a variety of common human diseases, and our goal has been to revitalize discovery made through genetic studies in rats. Due to its larger size and complex social behavior, the rat provides investigative opportunities that are implausible in the mouse. The rat has also been a useful model in aging studies, an area of expanding research activity.[2] We recently constructed a new rat reference genome, GRCr8 (2024), which added 179 Mb of sequence to the prior rat reference assembly (mRatBN7.2) and greatly improved assembly contiguity.[3]

The human genome telomere-to-telomere (T2T) project has demonstrated that ultra-long (UL)-read (Oxford Nanopore) genome sequencing yields assemblies that are both essentially complete and highly accurate.[4] We have adopted these approaches to generate a T2T assembly of one of the 8 inbred rat strains we previously assembled using HiFi sequencing (Pacific Biosciences) to provide as complete and accurate a representation of the rat genome as is currently achievable. We have used this assembly to provide a backbone of a rat pangenome to enhance comparative genome analysis across multiple rat strains. Critically, we have generated the new assembly from solid somatic tissues rather than cell lines that have been used for T2T assembly in humans and mice.[5–7] Cell lines can compromise assembly correctness due to non-episomal viral integration and somatic recombination within immune receptor genes,[8,9] and established lines may include latent chromosomal abnormalities or lack a full species chromosomal complement when cell lines are derived from female specimens.[4]

RESULTS

Assembly metrics

We selected the inbred Wistar-derived SHRSP strain for our studies, recognizing that the Brown Norway strain from which GRCr8 was generated is genetically more remote from other inbred rat strains we have assembled, while SHRSP shares ancestry with several of the strains whose genomes we have assembled.[10,11] We previously described the generation of HiFi reads and Hi-C chromosomal conformation capture sequencing for the initial assembly of this strain.[12] To complete the T2T assembly, we obtained UL sequencing reads that provided 32× genome coverage when reads of >100 kb were considered and 86× coverage when all UL reads passing quality controls

This is an open access article under the CC BY license (http://creativecommons.org/licenses/by/4.0/).

Table 1. Assembled chromosome sizes (bp) in the SHRSP T2T assembly, comparison with HiFi assembly of the SHRSP genome, and size differences (delta) among chromosomes

Chr	SHRSP T2T (bp)	SHRSP HiFi (bp)	SHRSP T2T-HiFi delta (bp)
Chr1	273,974,617	272,637,813	1,336,804
Chr2	251,298,693	252,699,238	−1,400,545
Chr3	169,854,131	181,816,842	−11,962,711
Chr4	184,839,043	184,652,898	186,145
Chr5	173,011,413	171,408,533	1,602,880
Chr6	146,793,982	146,571,229	222,753
Chr7	144,522,229	144,391,427	130,802
Chr8	131,669,355	133,631,198	−1,961,843
Chr9	129,549,291	129,265,479	283,812
Chr10	113,562,567	111,012,210	2,550,357
Chr11	99,926,075	92,243,620	7,682,455
Chr12	53,701,673	54,596,112	−894,439
Chr13	109,399,695	109,369,687	30,008
Chr14	109,260,687	109,134,705	125,982
Chr15	107,424,553	106,763,423	661,130
Chr16	92,830,114	92,316,445	513,669
Chr17	94,106,407	93,459,823	646,584
Chr18	85,849,073	85,801,627	47,446
Chr19	72,640,497	64,261,396	8,379,101
Chr20	57,885,825	56,811,052	1,074,773
ChrX	162,183,458	159,029,366	3,154,092
ChrY	77,331,694	47,402,513	29,929,181
Total	2,841,615,072	2,781,407,916	60,207,156

Table 2. Overall assembly statistics for the SHRSP T2T assembly compared with the prior SHRSP HiFi assembly, determination of segmental duplications, and repeat content of assemblies determined by RepeatMasker using the Rattus_ancestral.fasta repeat database

Assembly statistics	SHRSP T2T	SHRSP HiFi	Difference (%)
Assembled bases (Gbp)	2.89	2.91	−0.65%
Unplaced bases (Mbp)	13.83	126.11	−89.03%
Gap bases (kbp)	45.07	1,605.31	−97.19%
Number of contigs	199	4130	−95.18%
Contig NG50 (Mbp)	131.67	50.48	160.84%
Segmental duplications			
% of genome in segmental duplications	1.91	1.37	38.85%
Segmental duplications bases (Mbp)	54.53	39.96	36.46%
No. of segmental duplications	29,916	22,036	35.76%
RepeatMasker			
Percentage of repeats (%)	40.28	40.11	0.42%
Repeat bases (Mbp)	1,144.64	1,122.78	1.95%
Long interspersed nuclear elements	549.82	538.48	2.11%
Short interspersed 2nuclear elements	155.03	154.27	0.49%
Long terminal repeats	238.49	232.59	2.54%
DNA	3.00	2.99	0.35%
Unclassified	100.44	99.16	1.30%
Simple repeat	84.13	81.73	2.94%
Low complexity	9.81	9.73	0.84%

(QC) were considered. The source tissue was kidney from newborn animals. This allowed us to verify male sex during tissue collection to ensure representation of the X and Y chromosomes (chrX and chrY) in the assembly. The bioinformatic pipeline used to complete the T2T assembly is outlined in Figure S1.

As the SHRSP strain has been inbred since the 1960s, our assembly produced a haploid autosomal genome. We assessed the presence of latent regions of heterozygosity in the genome by k-mer analysis using Meryl/Merqury.[13] We provide a plot of k-mer frequencies (Figure S2), with the sex chromosome k-mers decomposed from the overall assembly, to reveal a single sigmoidal distribution of all components, evidence that this strain is fully inbred and that a male haploid assembly provides a complete genomic representation. We estimated assembly quality with Merqury using Illumina, HiFi, and ONT reads. The quality value (QV) of the overall assembly is 65.0, and the QV of individual chromosomes ranges from 56.2 to 79.8 (Table S1). This new T2T assembly of the rat genome has a total chromosomal extent of 2.84 Gbp, with an additional 13.8 Mbp located in unassembled contigs. Sixteen of the 22 chromosomes assembled T2T, without gaps. When assembly gaps were present, they occurred in the nucleolar organizing regions containing highly repetitive rDNA arrays on chr3, chr11, and chr12. The repetitive rDNA genes generated complex tangles that could not be fully resolved by the assembler and were only partially resolved by manual curation. Another gap on chr12 lies in the highly repetitive 5S rRNA locus. Gaps on chr4, chrX, and chrY were flanked by extensive regions of simple repeat sequences. In contrast to these 7 gaps, the HiFi-based GRCr8 assembly has 164 gaps, with 31 of the 44 telomeres incorporated into the assembly. Tables 1 and 2 provide a full assembly comparison. Notable changes include the addition of 60 Mb overall to the assembled chromosomes; changes to chr3, chr11, and chr12; a large increase in the assembled chrY; and an additional sequence in chr19. Analysis of completeness of the assembly with conserved mammalian gene sequences used BUSCO[14] and Compleasm[15] software and the mammalia and glires v.12 orthoDB lineage datasets.[16] BUSCO identified 97.4% (mammalia) and 98.4% (glires) of the orthologs as complete. For Compleasm analysis with the same ortholog databases, these values were 99.6% and 99.8%, respectively.

The unincorporated contigs often contained incomplete, complete, or multiple copies of 45S pre-ribosomal RNA genes (105 of 170 contigs). One of the contigs contained multiple 5S ribosomal genes. Centromeric satellite repeats were present in 10 contigs. Six contigs contained potential protein-coding genes, among which three contained copies of disks large MAGUK scaffold protein 5-like (Dlg5l) and IQ domain-containing genes. These

CellPress
OPEN ACCESS

Table 3. Comparison of genes not lifted from GRCr8 to SHRSP with other rat genome assemblies

GRCr8 chr	GRCr8 position	SHRSP T2T	SHRB2	WKY	LH	LN	Dahl SR	F344	Total missing
Chr1	3,605,095–3,607,942	Raet1c	Y	Y	Y	Y	Y	Y	6
Chr1	3,799,819–3,804,800	Raet1l	Y	Y	Y	Y	Y	Y	6
Chr1	41,719,650–42,011,103	Cd99l3	Y	–	–	–	–	–	1
Chr1	217,705,721–217,727,698	Ms4a6b	–	–	–	–	–	–	0
Chr1	241,917,517–241,919,287	Ears2l1	Y	Y	–	–	–	–	2
Chr2	133,748,793–133,798,656	Tgap1l6	Y	Y	Y		Y	Y	5
Chr2	135,532,788–135,571,218	Tgap1l5	Y	Y	Y		Y	Y	5
Chr2	184,436,760–184,437,857	Tdpoz2l12	–	–	–	–	–	–	0
Chr3	29,877,410–29,881,649	Lcn4l3	Y	Y	Y	Y	Y	–	5
Chr3	92,896,731–92,896,833	Rnu6-107	Y	–	–	–	–	–	1
Chr3	137,933,643–137,933,714	Snord57	Y	Y	–	–	Y	Y	4
Chr4	18,205,616–18,302,345	Cd36	Y	Y	Y	Y	Y	Y	6
Chr4	99,460,304–99,461,194	Igkvl2	Y	–	–	–	–	–	1
Chr5	100,508,027–100,510,921	Fbxl18l1	Y	Y	–	–	Y	Y	4
Chr5	108,233,731–108,234,318	Ifna5	Y	Y	Y	Y	–	–	4
Chr5	108,312,478–108,313,549	Ifna2l1	Y	–	Y	Y	–	–	3
Chr5	134,160,281–134,170,942	Cyp4a2	Y	Y	Y	Y	Y	–	5
Chr6	139,370,269–139,370,865	Ighl13	–	–	–	–	–	–	0
Chr6	139,796,674–139,797,173	Ighl3	–	–	–	–	–	–	0
Chr7	831,393–850,897	Hsd17b6	Y	Y	Y	Y	Y	Y	6
Chr7	1,031,374–1,047,152	Prim1	Y	Y	Y	Y	Y	Y	6
Chr7	108,653,283–108,660,112	Cyp11b1	Y	Y	Y	Y	Y		5
Chr7	134,678,294–134,682,759	Krt6c	Y	–	–	–	–	–	1
Chr7	136,823,892–136,827,506	Mucl1	Y	–	Y	Y	Y	–	4
Chr7	136,908,167–136,918,262	Spt1	Y	–	–	–	–	–	1
Chr7	136,940,229–136,943,813	Mucl2	Y	–	–	–	–	–	1
Chr10	100,652,729–100,660,524	Igsf7	Y	–	–	–	–	–	1
Chr12	23,927,389–23,941,894	Pilrb1l4	Y	Y	Y	Y	Y	Y	6
Chr14	4,797,089–4,841,322	Abcg3l2	Y	Y	–	–	–	Y	**3**
Chr14	4,849,327–4,901,486	Abcg3l4	Y	Y	–	–	–	Y	3
Chr14	5,007,953–5,009,683	Ube2v1l1	Y	–	–	–	–	Y	2
Chr14	21,361,378–21,362,147	Ppial4d	–	–	Y	Y	Y	Y	4
Chr15	29,692,420–29,696,756	Trav9-2l2	Y	Y	–	–	–	–	2
Chr15	30,053,595–30,057,357	Trav6-1	Y	Y	–	–	–	Y	3
Chr15	30,170,056–30,171,634	Trav14d-3-dv8l2	Y	Y	–	–	–	–	2
Chr15	30,765,617–30,766,193	Travl-ps2	Y	Y	–	–	–	–	2
Chr15	31,002,443–31,004,859	Trav9-2l1	–	Y	–	–	–	–	1
Chr15	37,568,092–37,592,492	Phf11	Y	–	–	Y	Y	Y	4
Chr15	38,953,398–39,084,298	Spata13	Y	–		Y	Y	Y	4
Chr16	11,189,677–11,191,907	Ralgdsl11	Y	–	Y	Y	Y	Y	5
Chr16	11,232,059–11,235,705	Cdrt15l2	Y	–	Y	Y	Y	Y	5
Chr17	14,661,166–14,667,821	Spata31c2l4	Y	Y	Y	Y	–	Y	5
Chr17	17,971,126–18,155,541	Kif13a	Y	–	–	–	–	–	**1**
Chr17	55,364,844–55,782,220	Hecw1	Y	–	–	–	–	–	**1**
Chr19	13,689,662–13,690,218	Trappc2ll1	Y	Y	Y	Y	Y	Y	6
Chr19	14,747,062–14,757,762	Speer4cl5	Y	–	–	–	–	–	1
Chr19	15,217,599–15,220,272	Dlg5l14	Y	–	–	–	–	–	1
Chr19	16,420,331–16,425,122	Dlg5l10	–	Y	–	–	–	Y	2

(*Continued on next page*)

Table 3. *Continued*

GRCr8 chr	GRCr8 position	SHRSP T2T	SHRB2	WKY	LH	LN	Dahl SR	F344	Total missing
Chr19	17,097,028–17,102,309	Ppp1cb-ps5	–	–	–	–	–	–	1
Chr19	17,695,332–17,700,102	Speer4cl6	–	–	–	–	–	–	0
Chr19	21,226,144–21,451,460	Iqcml1	Y	Y		Y	Y	Y	5
Chr19	21,358,733–21,361,239	Septin7-ps5	Y	Y	Y	Y	Y	Y	6
Chr19	23,062,094–23,064,466	Speer4cl4	–	–	–	–	–	–	0
Chr19	23,066,803–23,071,535	Dlg5l1	–	Y	–	Y	–	Y	3
Chr19	23,076,285–23,307,553	Iqcm	Y	Y	Y	Y	–	Y	5
Chr19	24,811,404–25,016,797	Speer4cl1	Y	–	–	–	–	–	1
Chr19	26,043,012–26,047,075	Dlg5l4	–	Y	–	Y	–	Y	3
Chr19	39,386,639–39,391,486	Speer4cl7	Y	Y	–	–	–	Y	3
ChrX	45,224,080–45,224,426	Ykt6l1	Y	Y	–	–	–	–	2

Genes annotated in GRCr8 and their position but missing from the Liftoff annotation of SHRSP T2T assembly are indicated in the third column. We examined 6 other genome assemblies to determine whether such genes were also unannotated in those assemblies (indicated by Y) after the same Liftoff process. On average, genes unannotated in SHRSP T2T were unannotated in 3 other assemblies. NCBI assembly accession numbers: GRCr8: GCF_036323735.1, SHRB2: GCA_023515785.2, WKY: GCA_023515805.2, LH: GCA_045687965.1, LN: GCA_045687995.1, Dahl SR: GCA_045688005.1, and F344: GCA_041222355.1.

genes occur in multiple copies in several expanded regions of the genome.

Annotation and novel gene discovery

A detailed annotation of the GRCr8 reference genome has been performed by the National Center for Biotechnology Information (NCBI), which provides a resource for computational annotation of the SHRSP T2T assembly. We achieved this using Liftoff software.[17] We compared the feature count in the SHRSP annotation file with that in the annotation file for GRCr8 and found that the total features it contained represented 100.24% of those in the GRCr8 annotation. Liftoff identifies and annotates duplicated genes, and the small excess of features in the SHRSP T2T assembly may reflect the detection of such copies. We also examined the Liftoff output to identify known protein-coding genes annotated in GRCr8 but not annotated in the SHRSP T2T assembly. Table 3 indicates that 59 such genes were unmapped in the T2T assembly. To assess whether this reflects a defect in the assembly or ancestral variation, we also examined Liftoff gene annotation in reference-quality genome assemblies of 6 other inbred rat strains. We found that only 6 genes in the T2T assembly were uniquely unmapped; most were unmapped in multiple strains, indicating that ancestral variation is likely to explain the absence of these genes.

We sought novel genes in the assembly that were unannotated by Liftoff because they are absent from the GRCr8 annotation. We used BRAKER3 software to combine GeneMark ETP, Augustus, and Tsebra gene prediction[18] with a collection of PacBio long-read RNA sequencing (RNA-seq) data from 19 distinct tissues of adult male and female animals of the SHRSP strain. This allowed us to identify annotations made only by BRAKER3 and absent from the Liftoff annotation. Alignment of IsoSeq data to the assembly allowed identification of open reading frames in regions annotated only by BRAKER3. Aligned transcript sequences were *in silico* translated, followed by a search of the NCBI Blastp database to identify homology between transcript sequences and known proteins. The rubric for calling potential novel genes is provided in Methods S1. This approach identified 65 plausible new coding sequences, listed in Table 4, including both novel genes and novel unannotated extensions of previously identified genes. Many were associated with structurally complex genomic regions known to contain strain-specific variation, including the immunoglobulin heavy chain (IGH) locus and the major histocompatibility complex (MHC) locus. We sought additional novel coding sequences by searching for genomic regions containing neither Liftoff nor BRAKER3 annotations but for which IsoSeq alignments suggested transcription of potential coding sequences. This yielded a further 26 potential coding genes with notable clusters of related genes on regions of chr15 and chr19 (Table 5).

Telomeres

All telomeres are represented in the assembly. Average telomere length in the assembly was 34.3 kb (SD = 34.0 kb), which corresponds well with previously estimated average telomere sizes in the rat[19] and mouse.[20] The high individual variation in length (1.4–121 kb) is similar to lengths obtained by targeted long-read telomere sequencing for mice (3–60 kb).[21]

We investigated telomere structure further by modifying the telomere profiling method applied to human telomeres and recently described by Karimian and colleagues.[22] This approach provides an estimate of the median telomeric repeat read length as well as read length distributions across multiple reads per chromosome arm. The mean telomeric length estimated this way showed less variation than was observed in the assembly (mean ± SD: 32.2 ± 6.2 kb). The average maximum telomere read length using this approach was 85.7 ± 25.4 kb. Estimating human telomere lengths this way reveals maximum lengths around 15 kb. Rat telomeres may be longer than the mean estimated using the telomere profiling approach. Length estimates in the rat may be constrained by the ability of nanopore sequencing to generate complete end-to-end subtelomere-to-telomere reads across the rat's longer telomeres. Telomere size estimates are provided in Table S2.

CellPress
OPEN ACCESS

Table 4. Novel genes not annotated by Liftoff but identified by BRAKER3 and having IsoSeq evidence of expression

Transcript span	Feature ID
Chr1:88,081,071–88,088,678	Kptn - addiitonal exons
Chr1:198,667,162–198,843,100	duplication of DMBT1 deleted in malignant brain tumors 1
Chr1:258,786,816–258,807,300	Sfxn, downstream exons
Chr2:138,837,468–138,855,544	stomatin-like protein 3, upstream exons
Chr2:233,930,000–233,974,055	guanylate-binding protein 4-like
Chr3:142,163,918–142,180,921	DNA methyltransferase 3C, partial - COOH end
Chr4:71,152,483–71,154,104	T cell receptor b
Chr5:137,426,283–137,429,290	Atp6v0b, upstream coding exons
Chr5:137,949,063–137,954,645	Med8, upstream coding exons
Chr6:138,333,842–138,339,200	immunoglobulin heavy chain
Chr6:138,495,190–138,496,842	immunoglobulin heavy chain
Chr6:138,525,547–138,535,647	immunoglobulin heavy chain
Chr6:138,618,792–138,628,952	immunoglobulin heavy chain
Chr6:138,678,486–138,683,476	immunoglobulin heavy chain
Chr6:138,712,170–138,722,289	immunoglobulin heavy chain
Chr6:138,775,218–138,776,863	immunoglobulin heavy chain
Chr6:138,805,578–138,815,655	immunoglobulin heavy chain
Chr7:116,295,850–116,299,462	Cyp11b1-related gene
Chr9:5,736,890–5,746,884	fibronectin type III and SPRY domain-containing protein 1, 3′ region novel exons
Chr9:6,767,415–6,777,049	adenylate kinase 1-like
Chr9:98,878,720–98,998,764	procollagen, type IV, alpha 4, isoform CRA_a
Chr10:20,334,552–20,337,028	serine protease 29-like
Chr10:108,282,915–108,293,521	St6galnac1 duplicate 1
Chr10:108,308,346–108,317,266	St6galnac1 duplicate 2
Chr11:50,499,529–50,526,034	duplication of Mx2
Chr15:21,620,452–21,630,252	disks large homolog 5-like
Chr15:38,382,636–38,512,256	spermatogenesis-associated protein 13
Chr16:19,320,347–19,335,178	downstream exons of armadillo repeat-containing protein 6
Chr17:17,708,394–17,858,979	kinesin-like protein KIF13A
Chr17:17,860,468–17,891,217	duplicate of kinesin-like protein KIF13A
Chr17:57,234,601–57,401,974	E3 ubiquitin-protein ligase HECW1
Chr17:61,071,818–61,074,351	ral guanine nucleotide dissociation stimulator-like
Chr19:192,724–201,350	pyrethroid hydrolase Ces2a precursor
Chr19:54,752,221–54,776,209	chymotrypsinogen B precursor, Ctrb1, duplicate 1
Chr19:54,812,289–54,815,718	chymotrypsinogen B precursor, Ctrb1, duplicate 2
Chr20:1,520,965–1,530,020	von Hippel Lindau-like
Chr20:2,766,272–2,771,323	MHC class I antigen-like (similar to RT1 class Ib, locus T18 precursor)
Chr20:3,395,338–3,402,865	similar to MHC class Ib alpha chain
Chr20:3,418,733–3,429,364	similar to MHC class Ib alpha chain
Chr20:3,493,013–3,497,118	similar to RT1 class I histocompatibility antigen, AA alpha chain precursor
Chr20:3,750,487–3,754,442	similar to RT1 class I histocompatibility antigen, AA alpha chain precursor
Chr20:3,764,751–3,772,682	similar to RT1 class I histocompatibility antigen, AA alpha chain precursor
Chr20:3,803,612–3,807,144	MHC class I antigen
Chr20:3,849,844–3,853,876	similar to RT1 class I, locus CE5 isoform
Chr20:3,864,755–3,872,880	similar to RT1 class I, locus CE13 isoform
Chr20:3,884,104–3,887,766	similar to RT1 class I histocompatibility antigen, AA alpha chain precursor-like
Chr20:3,910,186–3,914,121	similar to RT1 class I histocompatibility antigen, AA alpha chain precursor
Chr20:4,022,496–4,026,141	similar to RT1 class I histocompatibility antigen, AA alpha chain precursor

(*Continued on next page*)

Table 4. Continued

Transcript span	Feature ID
Chr20:4,120,787–4,124,304	similar to MHC class I antigen
Chr20:4,161,503–4,165,295	similar to RT1 class I, locus CE4 isoform
Chr20:4,175,598–4,179,221	similar to RT1 class I histocompatibility antigen, AA alpha chain
Chr20:4,197,121–4,200,739	similar to RT1 class I, locus CE1 isoform
Chr20:4,208,708–4,215,523	similar to RT1 class I histocompatibility antigen, AA alpha chain
Chr20:4,230,335–4,235,120	similar to RT1 class I, locus CE5 isoform
Chr20:4,283,486–4,291,254	similar to RT1 class I, locus CE13 precursor
Chr20:4,314,240–4,318,714	similar to MHC class Ib antigen
Chr20:4,796,499–4,829,943	tenascin XB precursor partial
Chr20:5,105,782–5,117,193	similar to butyrophilin-like 8
Chr20:5,137,987–5,148,248	similar to butyrophilin subfamily 3 member A2 precursor
ChrX:161,511,500–161,528,011	Dkc1x with additional downstream exons
ChrX:162,134,511–162,143,244	P55 erythrocyte membrane protein, Mpp1x
ChrX:162,149,757–162,167,993	Vbp1x
ChrY:19,027–37,638	Vbp1y
ChrY:43,647–52,652	P55 erythrocyte membrane protein, Mpp1y
ChrY:186,326–202,797	Dkc1y

Centromeres

The rat is intermediate between humans and mice regarding centromeric location. Human chromosomes are metacentric. Mouse chromosomes are telocentric and possess very large centromeric satellite arrays (up to 25–30 Mb) immediately adjacent to the telomere.[23] The rat possesses both telocentric and metacentric chromosomes.[24] To resolve whether telocentric chromosomes in the rat genome are analogous to mouse chromosomes in having long centromeres and whether karyotypically telocentric rat chromosomes are acrocentric, i.e., have non-repetitive sequence between the telomere and the nearby centromere, we used ModDotPlot software for initial identification of potential centromeric regions by analysis of chromosomal self-similarity.[25] Since centromeres comprise extended satellite repeats, self-similarity is expected to be high. Potential centromeres (Figure S3) identified using this method were located in positions suggested by rat cytogenetic studies.[24] However, some chromosomes were ambiguous in that more than one region of extensively repeated sequence was present. To distinguish regions containing centromeric satellite repeats, we annotated the assembly using RepeatMasker software and a database of known rat and ancestral rat repeat sequences available from the Genetic Information Research Institute.[26] We then examined the concurrence between annotation of rat centromeric satellite repeats and regions of high self-similarity. Figure 1 shows examples of a telocentric chromosome (chr4) and a metacentric chromosome with a single region of extensive self-similarity (chr13). Two additional metacentric chromosomes with additional regions of high self-similarity that do not reflect the location of the centromeric repeat sequences are shown (chr15 and chr19), highlighting non-centromeric processes that have given rise to extensive sequence duplication events. Focused views of the identified centromeres on each chromosome and the repeat annotations within and around them are shown in Figure S3. The annotated centromeres on chr1, chr3, chr19, and chrX diverge from the others in that they comprise adjacent blocks of centromeric repeat sequences interrupted by gaps of non-repetitive sequence. Notably, chr6–chr10 share structural similarity to telocentric mouse chromosomes with relatively large centromeres (4–6 Mb) lying immediately adjacent to the telomere. Studies in cultured human cells have identified a hypomethylated region of the centromere, the centromeric dip region, that is associated with active kinetochores.[27] However, somatic tissue from mouse lacked such regions,[28] which may be a feature of actively dividing cells.

Pseudo-autosomal gene-coding regions

The identification of pseudo-autosomal regions (PARs) on the rat sex chromosomes has been ambiguous.[29] However, our T2T assembly, in which both sex chromosomes concur with cytogenetic size estimates, provides an opportunity to investigate PARs in the rat sex chromosomes. ChrX and chrY in the rat are acrocentric.[24] The short p arm of chrY contains non-PAR coding sequences in which there has been extensive duplication of coding genes, including multiple copies of the sex-determining Sry and other genes (Figure 2). The region closer to the centromere also contains conserved single-copy genes, whereas few protein-coding genes are present in the Yq arm.

In many mammalian species,[30] including human and mouse, the PAR spans key genes required for development and immune regulation. Evolutionary pressure to conserve the function of essential genes within the PAR may provide the X and Y sequence similarity required for obligate synapsis, crossing over, and high-frequency recombination at this locus. However, our assembly of the GRCr8 rat reference revealed the absence of X-Y homology spanning gene-coding sequences at the p-arm termini of X and Y[3] (Figure 3A). We verified the genomic location of PAR genes that are present in the human chrY PAR region in our SHRSP T2T assembly and confirmed in the rat that 12 were autosomalized (i.e., identified on an autosome rather than a sex

CellPress
OPEN ACCESS

Table 5. Novel genes not annotated by Liftoff and not identified by BRAKER3 but having IsoSeq evidence of expression

Transcript span	Feature ID
Chr1:230,990,003–231,049,405	duplication of aldehyde dehydrogenase, cytosolic 1
Chr4:70,766,456–70,767,016	novel T cell receptor beta gene
Chr4:70,786,750–70,787,432	novel T cell receptor beta gene
Chr8:75,120,620–75,151,892	Aph1bl2, aph-1 homolog B, gamma secretase subunit-like 2
Chr12:10,866,476–10,909,575	duplicate copy of Ralbp1l1, ralA binding protein 1-like 1
Chr12:23,937,258–23,961,854	NXPE family member 3-like, neurexophilin and PC-esterase domain family, member 5-like 3
Chr12:24,045,683–24,070,431	duplication of NXPE family member 5-like 3, neurexophilin and PC-esterase domain family, member 5-like 3
Chr12:24,794,104–24,809,299	similar to paired immunoglobulin-like type 2 receptor alpha isoform X4
Chr13:77,574,802–77,598,630	similar to PRRC2B proline rich coiled-coil 2B
Chr13:77,593,395–77,605,852	partial rCG46573, isoform CRA_a, GenBank: EDM09397.1, potential PRR2C2 gene
Chr13:77,599,890–77,643,769	rCG46573, isoform CRA_a, GenBank: EDM09397.1, potential PRR2C2 gene
Chr13:88,566,388–88,584,759	similar to *Rattus rattus* interferon-activable protein 203-like
Chr14:4,486,493–4,523,820	similar to Abcg3l2 ATP-binding cassette, subfamily G (WHITE), member 3-like 2
Chr14:20,987,805–21,017,249	duplicate of UDP-glucuronosyltransferase 2B2
Chr15:20,795,098–20,805,297	copy of disks large homolog 5-like
Chr15:20,899,382–20,919,855	copy of disks large homolog 5-like
Chr15:21,105,413–21,112,812	copy of disks large homolog 5-like
Chr15:21,583,121–21,596,101	copy of disks large homolog 5-like
Chr15:21,716,520–21,726,648	copy of disks large homolog 5-like
Chr17:51,700,436–51,833,708	duplicate of amphiphysin, Amph
Chr19:15,096,100–15,119,559	copy of disks large homolog 5-like
Chr19:17,941,886–17,965,501	copy of disks large homolog 5-like
Chr19:17,941,886–17,965,501	copy of disks large homolog 5-like
Chr19:25,508,402–25,518,880	copy of disks large homolog 5-like
Chr19:25,995,006–26,012,663	copy of disks large homolog 5-like
Chr19:28,104,463–28,130,628	copy of disks large homolog 5-like

chromosome), 1 was present on the non-PAR region of the q arm of chrX, and 3 were absent from the assembly (Table S3). These observations add to a growing number of reports evidencing the rapid rate of structural variation and rearrangement of the mammalian PAR.[29,31]

Sex chromosome meiosis: Sequence analysis

Early studies examining sex chromosome meiosis in the rat proposed that chrY synapses throughout most of its length with X and that the short arm of X pairs with the long arm of Y.[32] Consistent with these findings, we observe high sequence similarity (>98%) between the proximal p-arm region of X and the distal q arm of Y. This region extends from the telomeric repeats over the adjacent 0.5 Mb of these two regions. Intriguingly, this similarity arises from arrays of repeated similar sequence (Figure 3B). While completely gene-free PARs appear to be rare or altogether absent in eutherian mammals, we note that there is precedent for our observation. Notably, the chicken PAR lacks protein-coding genes and exclusively comprises repetitive DNA,[33] and PARs in monotremes also lack coding sequences.[34]

Our survey of sequence homology between the sex chromosomes revealed a second block of X-Y homology (88%–90% identity) on chrX:4–6 Mb and chrY:69.5–73.5 Mb. This region harbors a multi-megabase expanse of 91ES8_RN centromeric satellite arrays on both chromosomes, with the chrX locus coinciding with the expected location of the chrX centromere. Repeat annotation reveals that chrY also has a second distinct array of SATI_RN and ISAT_RN satellite repeats at the junction of the short p arm and longer q arm (Figure 3B). Cytological data point to this proximal satellite array on Yp as the site of primary constriction and the functional centromere,[24] suggesting that the more distal chrY:69.5–73.5 satellite region is either an extinct centromere or a nonfunctional satellite array. Regardless, the discovery of a repetitive region on Yq that is homologous to the X centromere in sequence content and outside the presumptive PAR is novel. Nonfunctional, repeat-rich regions on the non-recombining chrY are subject to rapid evolution. Although speculative, occasional inter-chromosomal gene conversion between similar Xp and Yq satellite sequences could help preserve sequence homology, counter-balancing the mutational decay.

Sex chromosome meiosis: Cytogenetic analysis

We used cytogenetic analysis to directly visualize meiotic associations between the rat sex chromosomes. Synapsis occurs between the p arm and proximal q arm of chrX and extends across the majority of the q arm of chrY (Figure 3C). This chromosomal configuration places the distal chrY satellite array in proximity to the centromere of chrX, while centromeric proteins clearly

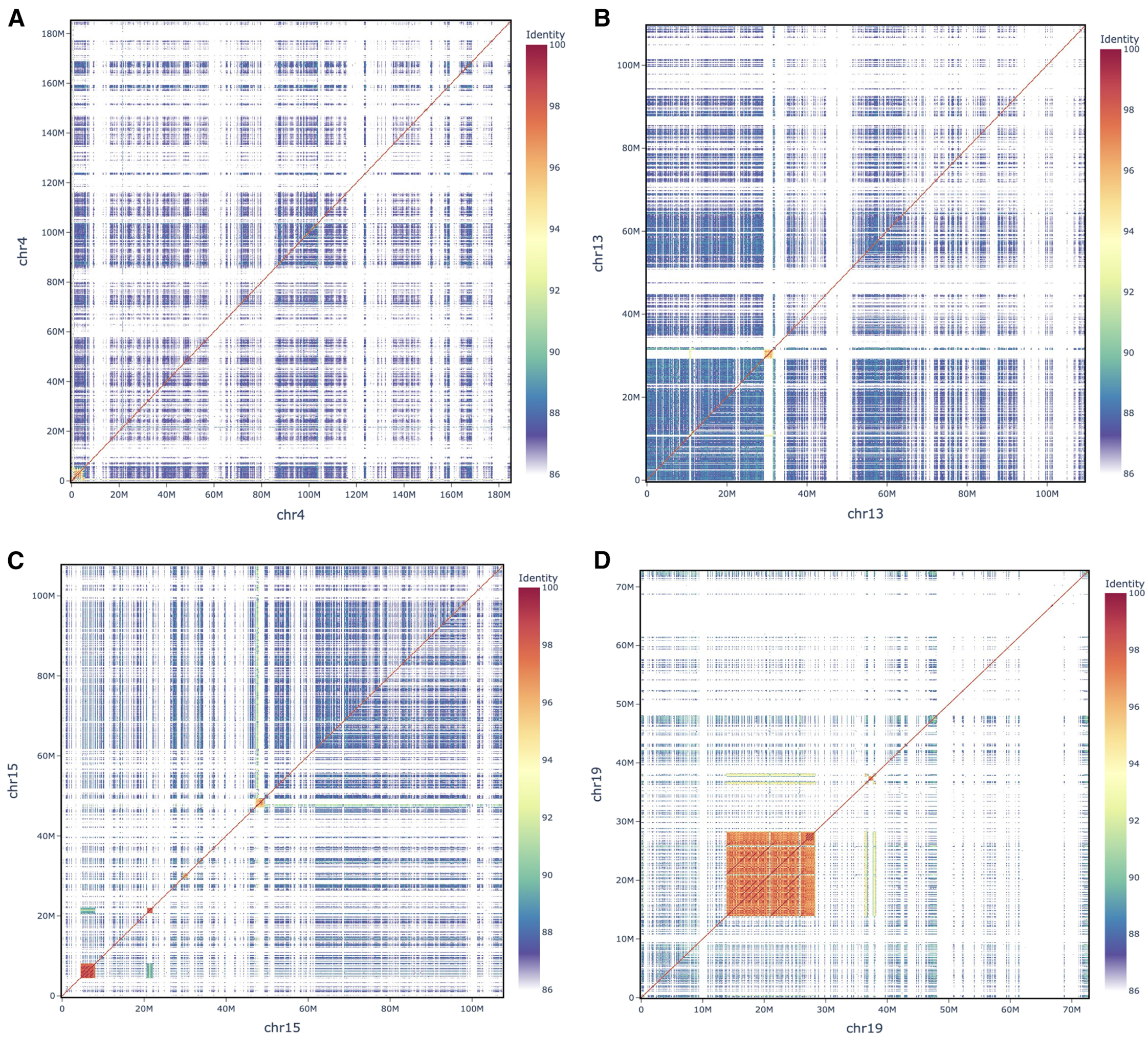

Figure 1. Centromeres comprised of tandem duplication of satellite sequences can be visualized by examination of sequence self-similarity
To aid in centromere identification, we used ModDotPlot software to visualize extended regions of self-similarity, shown here for chr4 (telocentric), chr13 (submetacentric), chr15 (metacentric), and chr19 (metacentric). Other regions of self-similarity are observed, notably on chr19 and also on chr15. These constitute repetitive regions of duplicated sequence discussed in the text.

accumulate at the Yp centromere. Crossover repair foci are regularly detected at the extreme distal end of chrYq. These crossover events are presumably occurring within the ∼0.5 Mb block of high X-Y sequence identity at the Xp and Yq termini. Intriguingly, the striated pattern of sequence homology across this distal Yq region (Figure 3B) reveals a landscape of higher-order repeats that may offer multiple alignment paths to support an obligate X-Y crossover. This genome architecture may also promote a chromatin configuration favorable to the recruitment of DNA double-strand break factors, similar to the mo-2 minisatellite arrays that dominate the mouse PAR.[35] Taken together, our findings indicate that the rat PAR is located at the terminal end of Yq and Xp and comprises repetitive DNA. Our findings also raise the possibility that homology between the X centromere and an inert Yq centromeric satellite array could facilitate X-Y homology recognition and/or synaptic associations in rat. This is schematized in Figure 3D.

Autosomal repeat regions: Functional implications

Several chromosomes (chr3, chr7, chr9, chr15, chr16, chr19, chr20, and chrY) contain regions of non-centromeric, highly repetitive sequence. Examples of such repetitive regions are presented in Figures 1C and 1D and, in total, extend across approximately 38 Mb of the assembly. Several of these regions contained multiply duplicated copies of coding sequence for genes that have been linked to sperm function, including members of the Spetex (sperm and testis expressed), Dlg5l (also known as spermatogenesis-associated glutamate-rich protein [SPEER]), sperm motility kinase W, IQCM (IQ motif-containing M), and POTEM (prostate, ovary and testis expressed M-like). A recently reported mouse T2T genome assembly also identified previously unassembled repetitive genomic regions containing some of these and related genes.[7]

A quantitative assessment of the duplicated region shown in Figure 1C on chr15:4.6–8.1 Mb indicated the presence of

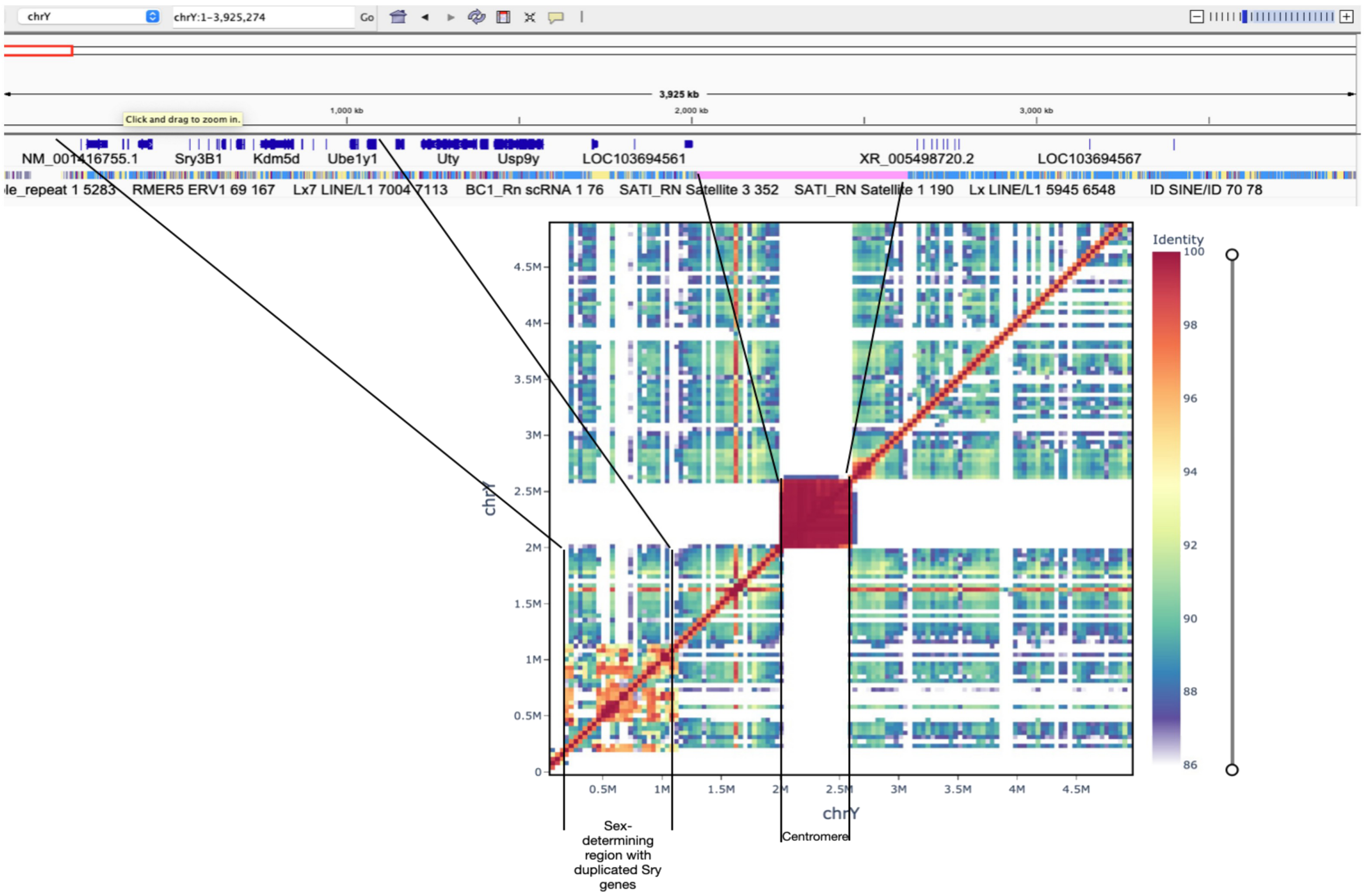

Figure 2. Repetitive sequence at the telomeric end of the chrY p arm

The top part of the figure shows the gene and repeat annotation tracks at proximal chrYp. The pink color indicates annotation of centromeric satellite sequences. Self-similarity across this region, indicated by ModDotPlot, is inserted below. The centromeric region is highly self-similar as expected (red square). There is also significant self-similarity in the sex-determining region of proximal chrY, reflecting the occurrence of gene duplication affecting the sex-determining Sry gene and other genes in the region.

interspersed multiple copies of Spetex family and disks large homolog 5-like genes in this region (21 and 37 genes annotated, respectively). Figure 1C reveals a second small block of self-similarity centered at 21 Mb. This block contains a further 20 annotated copies of disks large homolog 5-like genes, and our *de novo* annotation adds a further 5 copies (Table 5). A similar analysis of the duplicated region shown in Figure 1D on chr19:14–28 Mb indicates that Liftoff annotated 150 genes with the RefSeq description “disks large homolog 5-like” (SPEER genes) in this region. Our own *de novo* gene discovery (Table 5) identified a further six copies of these genes in this region.

Sex chromosome transmission distortion (STD) results in skewing of the sex ratio at birth.[36] STD effects become apparent when crosses are made between diverse inbred mouse strains but are not apparent within inbred strain matings.[36] Trait mapping in recombinant inbred mouse lines demonstrated STD but did not identify any loci of large effect.[36] Such effects might be difficult to detect if the mouse contains a similar number to rats of repetitive expanded autosomal loci that may affect sex chromosome transmission, as these duplicated genomic regions are challenging to adequately investigate by mapping. Moretti and colleagues have shown that amplification of Sycp3-like genes on chrX and chrY in the mouse drives a transcriptional response that contributes to STD by acting on transcriptional control of SPEER genes.[37] While it is unproven that duplicated genes in the rat that show biased sperm expression can create STD, these highly expanded genomic regions are strong candidates for such effects and can now be properly accounted for in studies to identify the biological basis of STD.

Autosomal repeat regions: Mapping implications

The incorporation of numerous regions containing duplicated sequence in our assembly can affect variant calling and genotyping, as reads from these regions cannot be mapped uniquely in the genome and are given a mapping quality of zero (MQ = 0). Variant calling and genotyping using GATK best practices will not analyze MQ = 0 reads. As such, there are regions in this assembly that are effectively invisible for the purposes of variant analysis in genome-wide association studies. We have identified these regions by looking for distances >100 kb between adjacent variants in a cohort VCF file called from short-read data of 8 distinct inbred rat strains. In Table S4, we provide a list of these regions in the SHRSP T2T genome that are >100 kb. In total, these regions comprise 17.4 Mb of the SHRSP T2T genome. The number 100 kb was chosen arbitrarily but is consistent with the lower bound used for analysis of runs of homozygosity.

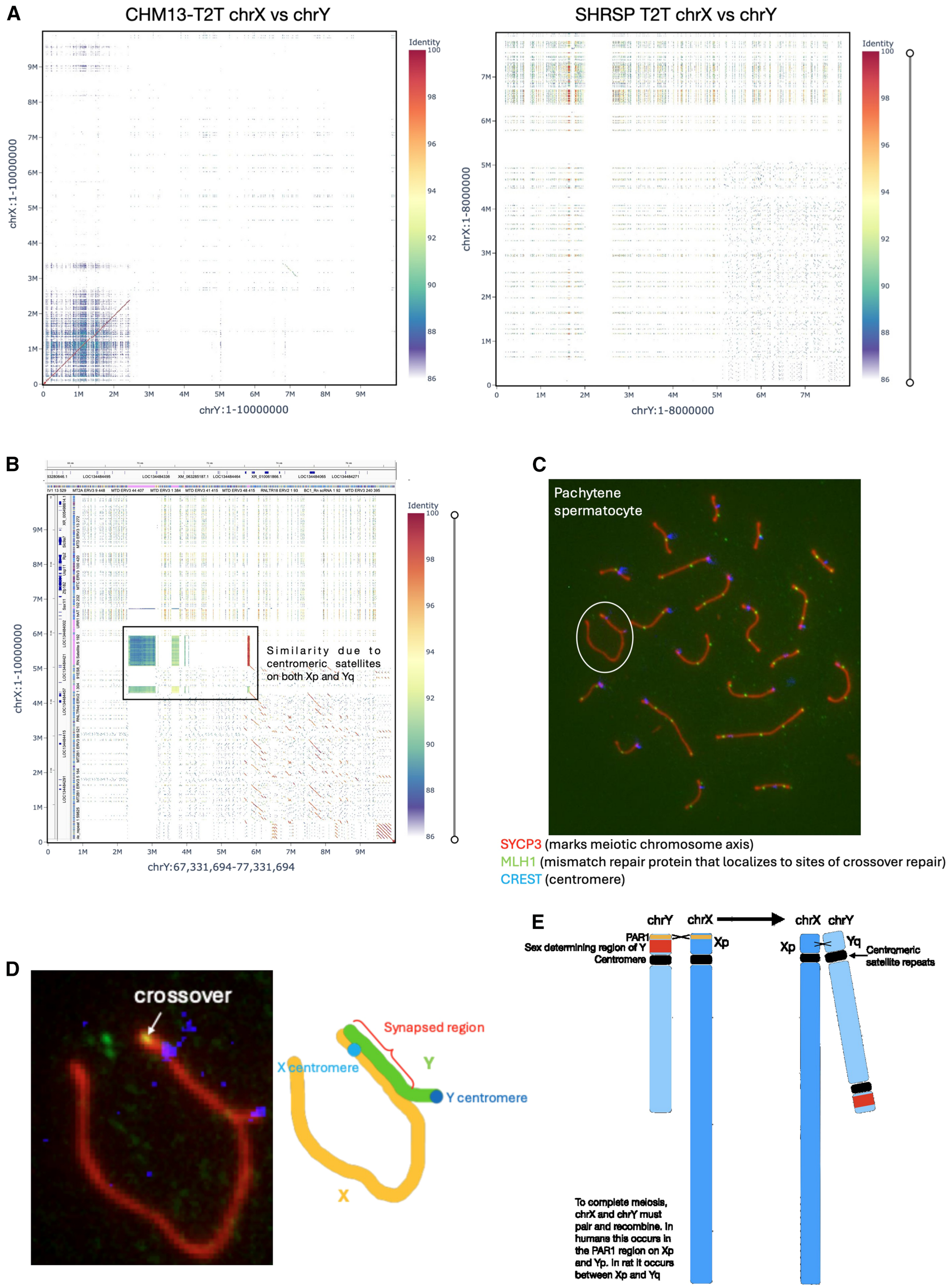

Figure 3. Sex chromosome pairing and meiosis in the rat

(A) PAR1 region of X and Y shows sequence similarity in humans but not in rats.

(B) Unexpectedly, distal chrY has extensive centromeric satellite sequence about 7 Mb upstream of the q-arm telomere. This cryptic centromere may be involved in synapsis and recombination. The inactive centromere in distal chrY is homologous to the proximal centromere of chrX and comprises the same centromeric

(legend continued on next page)

More regions would be identified with a smaller length threshold. We also count the total number of reads mapped to the region and provide a count of reads for the region with MQ > 0. In nearly all cases, the total read counts are consistent with the coverage seen elsewhere in the genome, while the MQ > 0 read counts are few, indicating that there are insufficient MQ > 0 mappings to call variants. In downstream analysis of the BAM file, this may lead to an inaccurate assessment of runs of homozygosity or erroneously suggest a highly conserved region resistant to variation. In Figure S4, we provide a screenshot of an IGV session for one of these regions showing unshaded MQ = 0 reads. In the STAR methods, we provide a set of scripts that can be used to identify regions affected by this artifact of arbitrary length consistent with the interests of any researcher. It is suggested that if there is a specific region of interest, it should be evaluated using a cohort genotype dataset with the provided scripts to determine whether variants and genotypes can be called in that region. We performed a similar analysis on the corresponding SHRSP HiFi-based assembly (GCA_021556685.1) and see that the number of these regions decreased markedly from 17.4 to 3.3 Mb. This is attributable to the duplication events responsible for this artifact being more faithfully and completely represented in the T2T assembly. These regions are enumerated in Tables S4 and S5.

Pangenome analysis of inbred rat strains: Pangenome creation

Pangenome graphs constructed from multiple high-quality genome assemblies can mitigate the bias associated with a reference genome assembly.[38] We generated a pangenome graph with SHRSP_T2T as the backbone and including seven reference-quality assemblies from additional rat strains using Minigraph-Cactus.[39] The pangenome is a useful tool for revealing structural variation in the rat genome. The raw VCF file was normalized with vcfbub to extract top-level variants, then filtered to retain structural variants (SVs) between 50 bp and 500 kb. SV lengths were derived from the difference between the reference and the longest alternate allele. Custom Python scripts parsed the graph path allele transversion tag (AT tag) annotations to classify each SV as insertion (INS), deletion (DEL), inversion (INV), or complex. SV genotypes were extracted to quantify the number of strains carrying each variant.

Pangenome analysis: Assessment of structural variation

SVs were binned into 500 kb windows across the genome to compute SV density per chromosome. Genomic regions with consistently high SV density were identified using a sliding-window approach based on a count threshold. Known immune gene loci that are expected *a priori* to be highly structurally variable (e.g., IGH, MHC, and T cell receptor alpha [TCRα]) were annotated. Separately, the SHRSP genome annotation file was used to calculate gene density in 500 kb windows. Both SV and gene densities were integrated into a single ideogram-like visualization to facilitate side-by-side comparison (Figure 4C).

Among all SVs, insertions were the most prevalent by event count (∼60%), followed by deletions (∼29%), with fewer inversions and complex events (Figure 4A). Length distribution analysis revealed a strong enrichment of insertions between 150 and 250 bp, consistent with SINE (short interspersed nuclear element) retrotransposon activity. A second peak of insertions at 6–7 kb, matching expected lengths of LINE (long interspersed nuclear element)-1 insertions (Figure 4B). Deletions showed a broad size distribution from 50 bp up to tens of kilobases, without clear peaks, reflecting diverse deletion mechanisms. Inversions and complex SVs were less size specific and scattered across scales. These findings emphasize the dominant role of mobile element insertions in shaping rat genome diversity.

SVs can be detected readily in a pangenome. With the pangenome graph, we can test the hypothesis that SV density would be lower in gene-rich regions because purifying selection in these regions inhibits the accumulation of SVs. In Figure 4C, it is shown that SV density differed markedly across chromosomes. For instance, chr5, chr12, and chr20 harbored regions with higher SV density, whereas chr9, chr11, and chr17 contained large contiguous segments in which no SVs were detected across all strains. Some such segments correspond to centromeric regions, while SV absence in other regions might reflect evolutionary constraint. To systematically define structurally conserved regions, we identified 1 Mb genomic bins in which all eight strains carried only the reference allele. Analysis of the full dataset showed a weak but statistically significant positive correlation (Spearman's rank correlation test, bin size: 100 kb) between SV density and gene density ($r = 0.14$, $p < 0.001$). This result suggests that SVs generally accumulate in regions of higher overall coding potential. When the analysis was restricted to testing the relationship within the extreme SV hotspots (>95% of SV count), the correlation was reduced ($r = 0.077$, $p = 0.00385$). The r value of 0.077 indicates that within the regions of extreme SV accumulation, the correlation between SV density and gene density approaches zero. This near-zero correlation statistically confirms our hypothesis that purifying selection dominates in these regions, effectively eliminating the expected positive correlation and ensuring that the most extreme SV accumulations are separated from gene-dense regions (Figure 4C). Code for this analysis is publicly available; see the key resources table in the STAR Methods for access to the

satellite repeat type. The terminal region of the chrY q arm and the terminal region of the chrXp arm share sequence similarity across 0.5 Mb. RepeatMasker annotations are shown adjacent to the axes. The pink-color sequence is the rat centromeric satellite sequence.

(C) Immunofluorescence analysis of pachytene chromosomal spread from rat spermatocytes. The synapsis between chrX and chrY is outlined in a white circle in the left image. The image is stained to identify the synapsis along the meiotic chromosome axis using SCYP3 antibodies (red). Centromere locations are highlighted in blue using an antibody to CREST. Mismatch repair protein indicates the location of crossover DNA break repair.

(D) The configuration of chrX and chrY is diagrammed, indicating that recombination occurs between the distal Yq arm and the proximal Xp arm.

(E) A model of sex chromosome meiosis in "human-like" sex chromosomes that possess a gene containing p-arm PAR on both the X and Y chromosomes compared to the rat sex chromosome meiosis in which there are no conserved PAR genes in the telomeric Yp arm and the orientation of chrY is flipped with respect to chrX. The putative neo-centromere in the Yq region is also indicated.

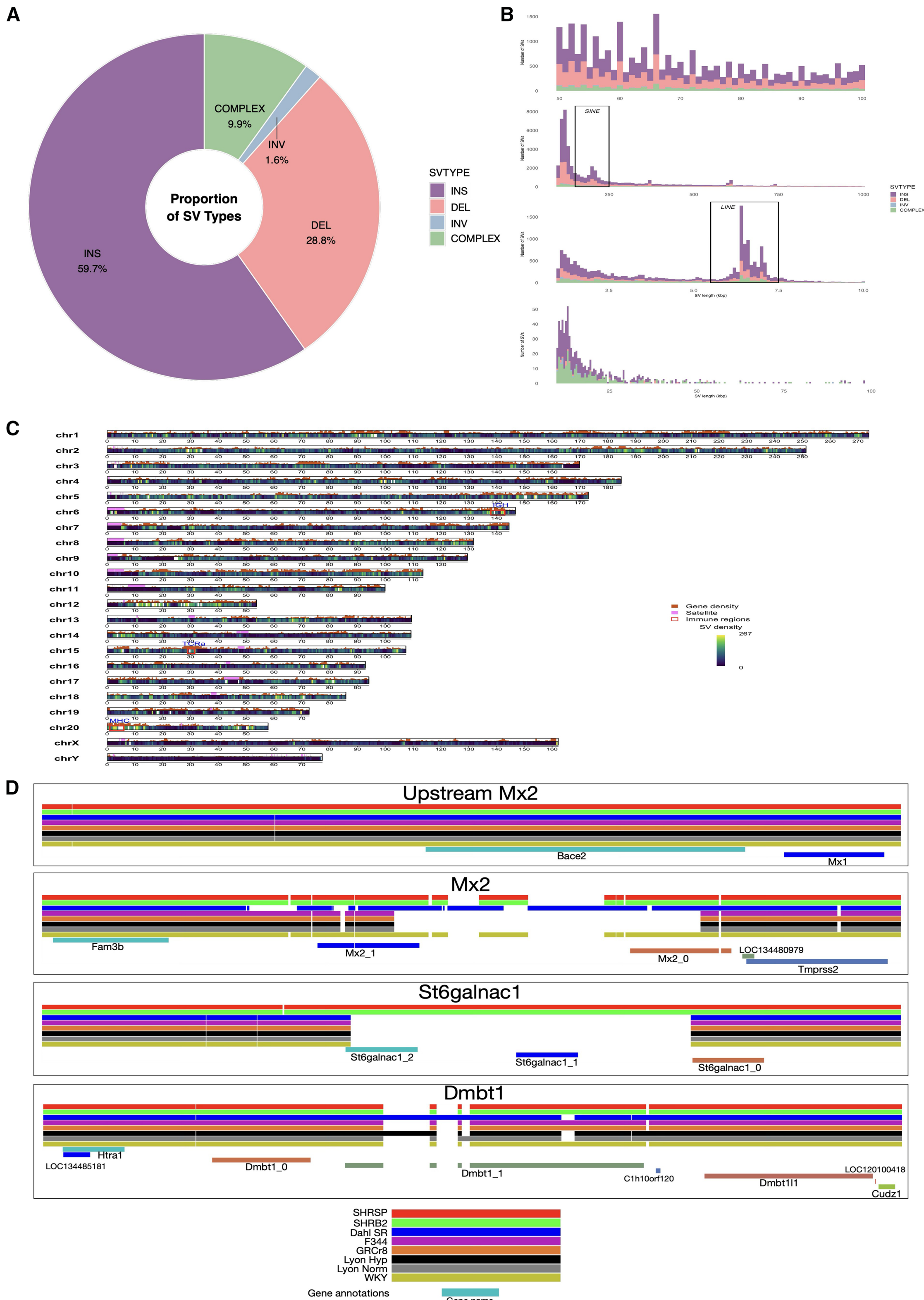

Figure 4. Global and local structural variation in the rat genome

(A) Proportion of pangenome structural variant (SV) distribution by event count. Percentages represent the proportion of each SV type's event count relative to the total number of SV events detected.

(legend continued on next page)

required VCF file and script. However, certain SV hotspots (e.g., on chr12 and chr20) did co-localize with high gene density, raising the possibility of regulatory impact or functional diversification. Several of these regions are highlighted in Figure 4C and represent the regions where immune response genes are found. These regions are known for hypervariability, and this finding suggests that SVs represent another way for a species to diversify its immune response, while other gene-rich regions appear not to benefit from this diversification, resulting in lower SV densities. We have performed a functional enrichment analysis of three co-localizing SV hotspot regions (on chr12 and chr20) using DAVID software.[40] The corresponding functional analysis generated by DAVID for these regions is provided in Data S1.

While retrotransposition undoubtedly is a source of abundant SVs in the rat genome, we have also examined the origins of SVs in gene-coding regions. Several of the newly annotated genes in this assembly represent events in which entire genes have been duplicated. Duplication in SHRSP affecting two genes involved in gut barrier function (Dmbt1 and St6galnac1) was observed, along with alignment of IsoSeq transcripts to both original and duplicated copies of these genes, suggesting adaptive value to increased gene dosage. Such duplications are likely the result of non-allelic homologous recombination (NAHR), and analysis of these regions supports gene-scale local sequence duplication.[41,42] Examples of genomic regions containing gene duplication events resulting from NAHR that are present in SHRSP but absent in GRCr8 are provided in Figure S5.

We observed clear SV enrichment at three major immune-related loci. The IGH locus, which spans >4 Mb on chr6, was dense, with complex insertions and inversions. The TCRα on chr15 was also enriched for SVs. The MHC on proximal chr20 is one of the most structurally variable regions across strains and reflects haplotype diversity that may give rise to inter-strain differences in immune response and disease susceptibility. These hypervariable loci play essential roles in antigen recognition, presentation, and adaptive immune responses occurring in T and B cells, and structural variation may accelerate adaptation to recently emerged pathogens.

To evaluate the ability of the pangenome assembly to represent genomic regions of structural complexity, we generated several visualizations of loci containing genes duplicated in SHRSP (Table 4) using ODGI software (Figure 4D).[43] Each illustration demonstrates structural variation occurring at the location where novel and duplicated genes were identified and shows how some such duplications may be present or absent in other strains. The images in Figure 4D represent 1D visualizations of complex variation across specific regions of the genomes of diverse strains. Our chief objective is to illustrate that such structural variation can be associated with gene duplication events and that this structural complexity can be revealed and investigated by simple images extracted from the pangenome graph. These illustrations also show that the T2T backbone on which the pangenome was structured can be integrated with our reference-quality HiFi-based genome assemblies from other strains, revealing highly consistent ancestral haplotypes shared by multiple strains.

Conclusions

The construction of a T2T genome assembly increases the extent, completeness, and accuracy of the assembled rat genome and permits the identification of many previously unrecognized genes. The assembly provides localization of complex repetitive structures such as telomeres and centromeres and brings to light several autosomal loci in which gene-encoding repetitive duplications are present. The assembly resolves the location and sequence composition of the PAR, revealing its higher-order repeat structure and the absence of genes. We further uncover an inactive centromere repeat array on Yq with homology to the X centromere and cytologically demonstrate that this locus undergoes heterologous synapsis during meiosis. Finally, the reference-quality assemblies of other inbred rat strains that we have generated permit the construction of a rat pangenome assembly from which insightful inferences regarding cross-genomic variation can be drawn, including in regions of great structural diversity.

Limitations of the study

The combination of sequencing and scaffolding methods used in this study sought to obtain the most complete and accurate possible representation of a rat genome. However, in addition to the gaps where contiguity could not be observed, the sequencing methods have intrinsic error rates that we have mitigated but not excluded.

The genome assemblies incorporated into the pangenome are reference quality but are unlikely to be as complete or accurate as the SHRSP-T2T assembly, as they lack nanopore UL reads.

We have presumed that the reported genealogies of the rat strains, which support full inbreeding, result in the presence of a haploid genome in each strain. We support this using k-mer analysis, which did not generate evidence to contravene this presumption; however, it cannot be fully proved, and the potential

(B) Size distribution of structural variation in the rat genome.

(C) Ideogram of the annotated genes and SV distribution. The distribution of annotated genes (orange bars at the top), satellite DNA (pink regions at the top), and immune-related regions (blue labels) are mapped across all chromosomes (chr1–chrY). The heatmap within each chromosome represents the density of SVs, with the color scale ranging from dark blue (low density, min = 0) to bright yellow (high density, max = 267). Genomic coordinates are indicated in Mb below each chromosome.

(D) Pangenome visualization of 4 loci of the genomes of 8 rat strains. These visualizations are centered on loci in which evidence of novel or duplicated genes was identified (see Table 4). In the case of the Mx2 locus, we also provide in the first image a visualization of a similar extent of the pangenome immediately upstream of the Mx2. This region largely lacks structural variation. In contrast, the three bottom images show structural variations that contain novel/duplicated genes in some strains. Much of the pangenome graph resembles the region immediately upstream of Mx2, in which there is minor structural variation. Each colored track represents a unique genome assembly as indicated in the bottom image. The upper track displayed in each image is the SHRSPT2T backbone assembly. Other tracks represent additional reference-quality genome assemblies of seven other rat strains, in descending order: SHRB2 (B2), Dahl salt resistant (DS), F344, Brown Norway (GRCr8), Lyon Hypertensive (LH), Lyon Normotensive (LN), and Wistar-Kyoto (WKY). The lower tracks are gene annotations, with primary gene annotations suffixed _0 and secondary _01 and _02.

for residual heterozygosity not reflected in the haploid assemblies remains.

Pangenome construction methods have yielded a pangenome that is reasonably consonant with expectations arising from the recent history of rat colonization of the Western Hemisphere and the genetic bottlenecks affecting the introduction of rats into the laboratory setting. However, a pangenome incorporating assemblies made from a broader source of Norway rats is necessary to develop a comprehensive rat species pangenome.

RESOURCE AVAILABILITY

Lead contact

Further information and requests for resources and reagents should be directed to and will be fulfilled by the lead contact, Peter A Doris, PhD (peter.a.doris@uth.tmc.edu).

Materials availability

The antibody reagents used were all commercially supplied and may be available from the vendors identified above. Experimental models are all inbred rat strains and may be obtained from the sites indicated. Not all strains may be available as live animals, but cryopreserved embryos are currently available for those that are not. Requestors will assume all costs associated with the supply of these models and will need to verify appropriate institutional approvals for animal research.

Data and code availability

All data and code are publicly available at the locations indicated or are described in the text.

ACKNOWLEDGMENTS

This work was supported by a grant from the National Institutes of Health to P.A.D., T.S.K., and M.L.S. (NHGRI R01HG011252). Work in P.A.D.'s lab is supported in part by the Mary Elizabeth Holdsworth Distinguished University Professorship.

AUTHOR CONTRIBUTIONS

Conceptualization, P.A.D., T.S.K., M.L.S., A.M.P., and S.K.; data curation, B.D.P.; formal analysis, P.A.D., T.S.K., K.L., and B.L.D.; funding acquisition, P.A.D., T.S.K., and M.L.S.; investigation, S.Y.B., M.L.S., K.L., T.S.K., and Y.Z.; methodology, K.L., S.K., W.A.L., G.G.B., and Y.Z.; project administration, P.A.D., M.L.S., and T.S.K.; resources, Y.Z.; software, S.K., K.L., and T.S.K.; visualization, B.L.D., K.L., T.S.K., and M.L.S.; writing – original draft, P.A.D., K.L., and T.S.K.; writing – review and editing, A.M.P., B.L.D., M.L.S., and S.K. All authors reviewed the results and approved the final version of the manuscript.

DECLARATION OF INTERESTS

The authors declare no competing interests.

STAR★METHODS

Detailed methods are provided in the online version of this paper and include the following:

- KEY RESOURCES TABLE
- EXPERIMENTAL MODEL AND STUDY PARTICIPANT DETAILS
- METHOD DETAILS
  - ONT-ultra-long sequencing
  - Long-read RNA sequencing
  - Genome assembly
  - K-mer analysis
  - Identification of unannotated novel genes
  - Repetitive sequence annotation
  - Identification of segmental duplications
  - Scripts for identifying duplicated regions resistant to short-read evaluation
  - Pseudo-autosomal region analysis
  - Pangenome graph construction and analysis
  - Structural variant analysis
- QUANTIFICATION AND STATISTICAL ANALYSIS

SUPPLEMENTAL INFORMATION

Supplemental information can be found online at https://doi.org/10.1016/j.xgen.2026.101281.

Received: October 14, 2025
Revised: March 25, 2026
Accepted: May 22, 2026
Published: August 6, 2026

REFERENCES

1. Gibbs, R.A., Weinstock, G.M., Metzker, M.L., Muzny, D.M., Sodergren, E.J., Scherer, S., Scott, G., Steffen, D., Worley, K.C., Burch, P.E., et al. (2004). Genome sequence of the Brown Norway rat yields insights into mammalian evolution. Nature *428*, 493–521.
2. Carter, C.S., Richardson, A., Huffman, D.M., and Austad, S. (2020). Bring Back the Rat!. J Gerontol A Biol Sci Med Sci. *75*, 405–415.
3. Li, K., Smith, M.L., Blazier, J.C., Kochan, K.J., Wood, J.M.D., Howe, K., Kwitek, A.E., Dwinell, M.R., Chen, H., Ciosek, J.L., et al. (2024). Construction and evaluation of a new rat reference genome assembly, GRCr8, from long reads and long-range scaffolding. Genome Res. *34*, 2081–2093.
4. Aganezov, S., Yan, S.M., Soto, D.C., Kirsche, M., Zarate, S., Avdeyev, P., Taylor, D.J., Shafin, K., Shumate, A., Xiao, C., et al. (2022). A complete reference genome improves analysis of human genetic variation. Science *376*, eabl3533.
5. Logsdon, G.A., Rozanski, A.N., Ryabov, F., Potapova, T., Shepelev, V.A., Catacchio, C.R., Porubsky, D., Mao, Y., Yoo, D., Rautiainen, M., et al. (2024). The variation and evolution of complete human centromeres. Nature *629*, 136–145.
6. Logsdon, G.A., Ebert, P., Audano, P.A., Loftus, M., Porubsky, D., Ebler, J., Yilmaz, F., Hallast, P., Prodanov, T., Yoo, D., et al. (2025). Complex genetic variation in nearly complete human genome. Nature *644*, 430–441.
7. Liu, J., Li, Q., Hu, Y., Yu, Y., Zheng, K., Li, D., Qin, L., and Yu, X. (2024). The complete telomere-to-telomere sequence of a mouse genome. Science *386*, 1141–1146.
8. Rodriguez, O.L., Sharp, A.J., and Watson, C.T. (2021). Limitations of lymphoblastoid cell lines for establishing genetic reference datasets in the immunoglobulin loci. PLoS One *16*, e0261374.
9. Lin, M.-J., Langmead, B., and Safonova, Y. (2025). IGLoo enables comprehensive analysis and assembly of immunoglobulin heavy-chain loci in lymphoblastoid cell lines using PacBio high-fidelity reads. Cell Rep. Methods *5*, 101033.
10. STAR Consortium; Saar, K., Beck, A., Bihoreau, M.-T., Birney, E., Brocklebank, D., Chen, Y., Cuppen, E., Demonchy, S., Dopazo, J., and et al.. (2008). SNP and haplotype mapping for genetic analysis in the rat. Nat. Genet. *40*, 560–566.
11. Munshi-South, J., Garcia, J.A., Orton, D., and Phifer-Rixey, M. (2024). The evolutionary history of wild and domestic brown rats (Rattus norvegicus). Science *385*, 1292–1297.
12. Kalbfleisch, T.S., Hussien AbouEl Ela, N.A., Li, K., Brashear, W.A., Kochan, K.J., Hillhouse, A.E., Zhu, Y., Dhande, I.S., Kline, E.J., Hudson, E.A., et al. (2023). The Assembled Genome of the Stroke-Prone Spontaneously Hypertensive Rat. Hypertens. Dallas Tex *80*, 138–146.

CellPress
OPEN ACCESS

13. Rhie, A., Walenz, B.P., Koren, S., and Phillippy, A.M. (2020). Merqury: reference-free quality, completeness, and phasing assessment for genome assemblies. Genome Biol. *21*, 245.
14. Manni, M., Berkeley, M.R., Seppey, M., Simão, F.A., and Zdobnov, E.M. (2021). BUSCO Update: Novel and Streamlined Workflows along with Broader and Deeper Phylogenetic Coverage for Scoring of Eukaryotic, Prokaryotic, and Viral Genomes. Mol. Biol. Evol. *38*, 4647–4654.
15. Huang, N., and Li, H. (2023). compleasm: a faster and more accurate reimplementation of BUSCO. Bioinforma. Oxf. Engl. *39*, btad595.
16. Zdobnov, E.M., Kuznetsov, D., Tegenfeldt, F., Manni, M., Berkeley, M., and Kriventseva, E.V. (2021). OrthoDB in 2020: evolutionary and functional annotations of orthologs. Nucleic Acids Res. *49*, D389–D393.
17. Shumate, A., and Salzberg, S.L. (2021). Liftoff: accurate mapping of gene annotations. Bioinforma. Oxf. Engl. *37*, 1639–1643.
18. Gabriel, L., Brůna, T., Hoff, K.J., Ebel, M., Lomsadze, A., Borodovsky, M., and Stanke, M. (2024). BRAKER3: Fully automated genome annotation using RNA-seq and protein evidence with GeneMark-ETP, AUGUSTUS, and TSEBRA. Genome Res. *34*, 769–777.
19. Gomes, N.M.V., Ryder, O.A., Houck, M.L., Charter, S.J., Walker, W., Forsyth, N.R., Austad, S.N., Venditti, C., Pagel, M., Shay, J.W., and Wright, W.E. (2011). Comparative biology of mammalian telomeres: hypotheses on ancestral states and the roles of telomeres in longevity determination. Aging Cell *10*, 761–768.
20. Prowse, K.R., and Greider, C.W. (1995). Developmental and tissue-specific regulation of mouse telomerase and telomere length. Proc. Natl. Acad. Sci. USA *92*, 4818–4822.
21. Smoom, R., May, C.L., Ortiz, V., Tigue, M., Kolev, H.M., Rowe, M., Reizel, Y., Morgan, A., Egyes, N., Lichtental, D., et al. (2023). Telomouse-a mouse model with human-length telomeres generated by a single amino acid change in RTEL1. Nat. Commun. *14*, 6708.
22. Karimian, K., Groot, A., Huso, V., Kahidi, R., Tan, K.-T., Sholes, S., Keener, R., McDyer, J.F., Alder, J.K., Li, H., et al. (2024). Human telomere length is chromosome end-specific and conserved across individuals. Science *384*, 533–539.
23. Francis B, Gozashti L, Costello K, Kasahara T, Harringmeyer OS, Lilue J, Helmy M, Kato T, Czechanski A, Quail MA, et al. The structural diversity of telomeres and centromeres across mouse subspecies revealed by complete assemblies [Internet]. 2024 [cited 2024 Dec 28];2024.10.24.619615. Available from: https://www.biorxiv.org/content/10.1101/2024.10.24.619615v2
24. Hamta, A., Adamovic, T., Samuelson, E., Helou, K., Behboudi, A., and Levan, G. (2006). Chromosome ideograms of the laboratory rat (Rattus norvegicus) based on high-resolution banding, and anchoring of the cytogenetic map to the DNA sequence by FISH in sample chromosomes. Cytogenet. Genome Res. *115*, 158–168.
25. Sweeten, A.P., Schatz, M.C., and Phillippy, A.M. (2024). ModDotPlot-rapid and interactive visualization of tandem repeats. Bioinforma. Oxf. Engl. *40*, btae493.
26. Bao, W., Kojima, K.K., and Kohany, O. (2015). Repbase Update, a database of repetitive elements in eukaryotic genomes. Mob. DNA *6*, 11.
27. Gershman, A., Sauria, M.E.G., Guitart, X., Vollger, M.R., Hook, P.W., Hoyt, S.J., Jain, M., Shumate, A., Razaghi, R., Koren, S., et al. (2022). Epigenetic patterns in a complete human genome. Science *376*, eabj5089.
28. Chaudhry, G., Chen, J., Snipes, L., Bahl, S., Packiaraj, J., Lin, X., and Thakur, J. (2025). Genomic and epigenomic maps of mouse centromeres and pericentromeres. Nat. Commun. *16*, 9688.
29. Maxeiner, S., Benseler, F., Krasteva-Christ, G., Brose, N., and Südhof, T.C. (2020). Evolution of the Autism-Associated Neuroligin-4 Gene Reveals Broad Erosion of Pseudoautosomal Regions in Rodents. Mol. Biol. Evol. *37*, 1243–1258.
30. Raudsepp, T., and Chowdhary, B.P. (2015). The Eutherian Pseudoautosomal Region. Cytogenet. Genome Res. *147*, 81–94.
31. Wilson, M.A., and Makova, K.D. (2009). Genomic analyses of sex chromosome evolution. Annu. Rev. Genomics Hum. Genet. *10*, 333–354.
32. Joseph, A.M., and Chandley, A.C. (1984). The morphological sequence of XY pairing in the Norway rat Rattus norvegicus. Chromosoma *89*, 381–386.
33. Bellott, D.W., Skaletsky, H., Cho, T.-J., Brown, L., Locke, D., Chen, N., Galkina, S., Pyntikova, T., Koutseva, N., Graves, T., et al. (2017). Avian W and mammalian Y chromosomes convergently retained dosage-sensitive regulators. Nat. Genet. *49*, 387–394.
34. Zhou, Y., Shearwin-Whyatt, L., Li, J., Song, Z., Hayakawa, T., Stevens, D., Fenelon, J.C., Peel, E., Cheng, Y., Pajpach, F., et al. (2021). Platypus and echidna genomes reveal mammalian biology and evolution. Nature *592*, 756–762.
35. Takahashi, Y., Mitani, K., Kuwabara, K., Hayashi, T., Niwa, M., Miyashita, N., Moriwaki, K., and Kominami, R. (1994). Methylation imprinting was observed of mouse mo-2 macrosatellite on the pseudoautosomal region but not on chromosome 9. Chromosoma *103*, 450–458.
36. Haines, B.A., Barradale, F., and Dumont, B.L. (2021). Patterns and mechanisms of sex ratio distortion in the Collaborative Cross mouse mapping population. Genetics *219*, iyab136.
37. Moretti, C., Blanco, M., Ialy-Radio, C., Serrentino, M.-E., Gobé, C., Friedman, R., Battail, C., Leduc, M., Ward, M.A., Vaiman, D., et al. (2020). Battle of the Sex Chromosomes: Competition between X and Y Chromosome-Encoded Proteins for Partner Interaction and Chromatin Occupancy Drives Multicopy Gene Expression and Evolution in Muroid Rodents. Mol. Biol. Evol. *37*, 3453–3468.
38. Paten, B., Novak, A.M., Eizenga, J.M., and Garrison, E. (2017). Genome graphs and the evolution of genome inference. Genome Res. *27*, 665–676.
39. Hickey, G., Monlong, J., Ebler, J., Novak, A.M., Eizenga, J.M., Gao, Y., Human Pangenome Reference Consortium; Marschall, T., Li, H., and Paten, B. (2024). Pangenome graph construction from genome alignments with Minigraph-Cactus. Nat. Biotechnol. *42*, 663–673.
40. Dennis, G., Sherman, B.T., Hosack, D.A., Yang, J., Gao, W., Lane, H.C., and Lempicki, R.A. (2003). DAVID: Database for Annotation, Visualization, and Integrated Discovery. Genome Biol. *4*, P3.
41. Höps, W., Rausch, T., Jendrusch, M., Human Genome Structural Variation Consortium HGSVC; Korbel, J.O., and Sedlazeck, F.J. (2024). Impact and characterization of serial structural variations across humans and great apes. Nat. Commun. *15*, 8007.
42. Carvalho, C.M.B., and Lupski, J.R. (2016). Mechanisms underlying structural variant formation in genomic disorders. Nat. Rev. Genet. *17*, 224–238.
43. Guarracino, A., Heumos, S., Nahnsen, S., Prins, P., and Garrison, E. (2022). ODGI: understanding pangenome graphs. Bioinforma. Oxf. Engl. *38*, 3319–3326.
44. Cheng, H., Concepcion, G.T., Feng, X., Zhang, H., and Li, H. (2021). Haplotype-resolved de novo assembly using phased assembly graphs with hifiasm. Nat. Methods *18*, 170–175.
45. Li, H. Seqtk: a toolkit for processing sequences in FASTA/Q formats. [Internet]. 2018;Available from: https://github.com/lh3/seqtk
46. Rautiainen, M., and Marschall, T. (2020). GraphAligner: rapid and versatile sequence-to-graph alignment. Genome Biol. *21*, 253.
47. Uliano-Silva, M., Ferreira, J.G.R.N., Krasheninnikova, K., Darwin Tree of Life Consortium; Formenti, G., Abueg, L., Torrance, J., Myers, E.W., Durbin, R., Blaxter, M., and McCarthy, S.A. (2023). MitoHiFi: a python pipeline for mitochondrial genome assembly from PacBio high fidelity reads. BMC Bioinf. *24*, 288.
48. Walker, B.J., Abeel, T., Shea, T., Priest, M., Abouelliel, A., Sakthikumar, S., Cuomo, C.A., Zeng, Q., Wortman, J., Young, S.K., and Earl, A.M. (2014). Pilon: an integrated tool for comprehensive microbial variant detection and genome assembly improvement. PLoS One *9*, e112963.
49. Astashyn, A., Tvedte, E.S., Sweeney, D., Sapojnikov, V., Bouk, N., Joukov, V., Mozes, E., Strope, P.K., Sylla, P.M., Wagner, L., et al. (2024). Rapid and

sensitive detection of genome contamination at scale with FCS-GX. Genome Biol. *25*, 60.

50. Li, H. (2013). Aligning sequence reads, clone sequences and assembly contigs with BWA-MEM. arxiv. https://doi.org/10.48550/arXiv.1303.3997.

51. Poplin, R., Ruano-Rubio, V., DePristo, M., Fennell, T., Carneiro, M., Van der Auwera, G., Kling, D., Gauthier, L., Levy-Moonshine, A., Roazen, D., et al.. Scaling accurate genetic variant discovery to tens of thousands of samples. https://www.biorxiv.org/content/10.1101/201178v3.

52. Antipov, D., Rautiainen, M., Nurk, S., Walenz, B.P., Solar, S.J., Phillippy, A.M., and Koren, S. (2025). Verkko2 integrates proximity-ligation data with long-read De Bruijn graphs for efficient telomere-to-telomere genome assembly, phasing, and scaffolding. Genome Res. *35*, 1583–1594.

53. Li, H. (2021). New strategies to improve minimap2 alignment accuracy. Bioinforma. Oxf. Engl. *37*, 4572–4574.

54. Išerić, H., Alkan, C., Hach, F., and Numanagić, I. (2022). Fast characterization of segmental duplication structure in multiple genome assemblies. Algorithms Mol. Biol. AMB. *17*, 4.

55. Dumont, B.L., and Payseur, B.A. (2011). Evolution of the genomic recombination rate in murid rodents. Genetics *187*, 643–657.

STAR★METHODS

KEY RESOURCES TABLE

REAGENT or RESOURCE	SOURCE	IDENTIFIER
Antibodies		
Rabbit polyclonal antibody against MLH1	Sigma-Aldrich	PC56
Goat polyclonal antibody against SYCP3	Santa Cruz Biotechnology	sc-20845; RRID: AB_2087203
Anti-human CREST antibody	Antibodies Inc	#15-235
Donkey anti-goat Rhodamine Red-X	Jackson ImmunoResearch	#705-295-003; RRID: AB_2340422
Donkey anti-mouse FITC	Jackson ImmunoResearch	# 715-095-150; RRID: AB_2340792
Donkey anti-human Coumarin AMCA	Jackson ImmunoResearch	# 709-095-149; RRID: AB_2340514
Experimental models: Rat (*Rattus norvegicus*) strains are available as indicated		
SHRSP/BbbUtx strain	Doris Lab	RGD_8142383
BN/NHsdMcwi	Med College Wisc	RGD_61498
F344/Stm	Med College Wisc	RGD_1302686
LN/MavRrrcAek	Med College Wisc	RGD_10755354
LH/MavRrrcAek	Med College Wisc	RGD_10755352
Dahl SR/JrHsd	Med College Wisc	RGD_1582184
WKY/Bbb	Doris Lab	RGD_1581635
SHR/Utx	Doris Lab	RGD_8142385
SHRSP Rat T2T genome assembly	NCBI SRA	PRJNA793432
HiFi sequencing data	NCBI SRA	PRJNA793432
ONT sequencing data	NCBI SRA	PRJNA793432
Hi-C sequencing data	NCBI SRA	PRJNA793432
Iso-Seq data	NCBI SRA	PRJNA793432
PacBio CLR sequencing data	NCBI SRA	PRJNA793432
Illumina WGS sequencing data	NCBI SRA	PRJNA793432
Pangenome graph data	This study Mendeley Data	Mendeley Data: https://doi.org/10.17632/7tydm36c54.2
Cytogenetic images	This study Mendeley Data	Mendeley Data:
Custom scripts	This study	Mendeley Data:
Trim Galore v0.6.10	Krueger	https://github.com/FelixKrueger/TrimGalore
Filtlong v0.2.1	Wick et al.	https://github.com/rrwick/Filtlong
Verkko v2.0	Rautiainen et al., 2023	https://github.com/marbl/verkko
hifiasm v0.19.8	Cheng et al.[44] 2021	https://github.com/chhylp123/hifiasm
seqtk v1.4	Li,[45] 2018	https://github.com/lh3/seqtk
GraphAligner v1.0	Rautiainen and Marschall,[46] 2020	https://github.com/maickrau/GraphAligner
BandageNG	Korobeynikov et al.	https://github.com/asl/BandageNG
MitoHiFi v3.2	Uliano-Silva et al.[47] 2023	https://github.com/marcelauliano/MitoHiFi
DeepPolisher v0.1.0	Google Research	https://github.com/google/deeppolisher
Pilon v1.24	Walker et al.[48] 2014	https://github.com/broadinstitute/pilon
FCS-GX	Astashyn et al.[49] 2024	https://github.com/ncbi/fcs
BWA-MEM	Li,[50] 2013	https://github.com/lh3/bwa
SAMtools	Danecek et al., 2021	http://www.htslib.org/
GATK v4.4	Poplin et al.[51] 2017	https://gatk.broadinstitute.org/
Meryl v1.4.1	Marçais et al.	https://github.com/marbl/meryl
Liftoff v1.6.3	Shumate and Salzberg,[17] 2021	https://github.com/agshumate/Liftoff
BRAKER3 v3.0.8	Gabriel et al.[18] 2024	https://github.com/Gaius-Augustus/BRAKER
RepeatMasker v4.1.8	Smit et al.	https://www.repeatmasker.org/
ModDotPlot	Sweeten et al.[25] 2024	https://github.com/marbl/ModDotPlot

(Continued on next page)

Continued

REAGENT or RESOURCE	SOURCE	IDENTIFIER
Minigraph-Cactus v8.1.0b1	Hickey et al.[39] 2024	https://github.com/ComparativeGenomicsToolkit/cactus
vcfbub v0.1.1	Garrison et al.	https://github.com/pangenome/vcfbub
R v4.5.1	R Core Team	https://www.r-project.org/
vcfR	Knaus and Grünwald	https://github.com/knausb/vcfR
ggplot2	Wickham	https://ggplot2.tidyverse.org/

EXPERIMENTAL MODEL AND STUDY PARTICIPANT DETAILS

For PacBio HiFi sequencing and Hi-C data generation we collected liver tissue from an 18 week old male SHRSP rat (Rat Genome Database identifier number RGD_8142383). For Oxford Nanopore sequencing, we collected kidney tissue from newborn rat pups of the same SHRSP strain. Animals were euthanized and sexed by opening of the abdominal cavity and identification of the presence of intra-abdominal testes. Only male animals were used in order to ensure equal representation of X and Y chromosomes in our assembly. Kidneys were collected and frozen in liquid nitrogen and stored at −80C prior to shipment to the sequencing core.

Tissue RNA for IsoSeq. We collected 19 distinct tissues from an 18 week old male animal of the same SHRSP strain. In addition, ovaries were collected from a female SHRSP of similar age. Tissues were placed into RNAlater and stored at −80°C prior to shipment to the sequencing core. All animal use was prospectively reviewed and approved by the Animal Welfare Committee (IACUC) of the University of Texas Health Science Center at Houston (approval ID# AWC-18-0147).

METHOD DETAILS

ONT-ultra-long sequencing

For ONT ultra-long sequencing, high molecular weight (HMW) DNA was extracted from single kidneys that had been chopped. We used the New England BioLabs Monarch T3060S kit and the manufacturer's protocol. DNA analysis was performed using Qubit fluorometric (ThermoFisher Scientific) measurement of extracted DNA. ONT Library preparation used the ONT sequencing kit #ULK001. Libraries were sequenced on a PromethION nanopore sequencer. A total of 12 samples were sequenced. Base calling employed either Dorado 7.0.9 or Dorado 7.2.13.

DNA preparation for PacBio HiFi sequencing and Hi-C chromosomal conformation sequencing was performed as previously described.[12]

Long-read RNA sequencing

Nucleated blood cells were purified by density gradient centrifugation using LymphoPrep (MPBio, Irvine, CA) and transferred to RNAProtect Cell Reagent (Qiagen). Tissue samples were collected into RNAlater (ThermoFisher) and frozen at −80°C prior to shipping on dry ice to the sequencing core. RNA was extracted from∼25 mg of tissue using the RNeasy Mini Kit (Qiagen), as recommended by the manufacturer. The quality and quantity of the RNA were assessed by a Bioanalyzer 2000 (Agilent) and Qubit fluorometry, respectively. Initial tissue samples were analyzed using the PacBio Iso-Seq pipeline on the PacBio IIe sequencer. After it became available, further samples were sequenced using the Kinnex pipeline on the PacBio Revio sequencer. Barcoded Iso-Seq SMRTbell libraries were generated as recommended by the manufacturer (Pacific Biosciences) and sequenced using 2.1 chemistry and 30 h movies on the Sequel IIe system. Prior to sequencing, RNA samples were multiplexed in batches of four tissues, and each fourplex pool was sequenced using a single SMRTcell. Following data collection, HiFi reads were generated on the instrument and used as input into the Iso-Seq v3 workflow within the SMRT Link v10.0 online SMRT sequencing bioinformatics suite. In total 19 unique tissues were sampled by long read RNA sequencing. The data was processed to generate a high quality BAM file for each tissue in which only unique isoforms were represented. The BAM files generated were converted to FASTA files and aligned to the genome assembly using minimap2. The resulting FLNC sam file was converted to BAM format using SAMtools, aligned to the genome assembly using minimap2, sorted, and indexed for viewing in Integrative Genomics Viewer (IGV) software.

Genome assembly

Raw Arima-C and Illumina WGS reads were adapter- and quality-trimmed using Trim Galore (v0.6.10) with default settings. PacBio HiFi reads were filtered using Filtlong to retain only reads with minimum quality ≥20 and length ≥1 kb. ONT ultra-long reads were also filtered with Filtlong, discarding reads with quality <10.

Genome assembly was performed using both Verkko (v2.0)[52] and hifiasm (v0.19.8),[44] using the same set of filtered reads. We selected the Verkko assembly for downstream analyses based on assembly continuity and accuracy metrics evaluated with seqtk.[45] Hi-C reads were aligned using the Arima mapping pipeline, and the resulting contact maps were used to correct mis-joins and scaffold breaks by gap insertion and contig reordering. Filtered ONT reads and the hifiasm assembly were aligned back to the Verkko assembly using GraphAligner (v1.0),[46] and Bandage NG was used to manually resolve tangle regions and fill gaps. Path files guided

by Bandage NG, together with supporting node-link files, were used as input for a further round of Verkko to generate the finalized assembly.

The mitochondrial genome was separately assembled using MitoHiFi,[47] and the circularized mitochondrial sequence was inserted into the nuclear assembly to prevent over-polishing of nuclear mitochondrial segments (NUMTs) during polishing steps.

Polishing was conducted using DeepPolisher (v0.1.0) with PacBio HiFi reads, followed by Pilon (v1.24)[48] with Illumina short reads. To eliminate residual contamination, final assemblies were screened using the NCBI Foreign Contamination Screening (FCS) toolkit.[49]

K-mer analysis

We developed an analytical pipeline following an approach outlined in recent papers.[50,51,53] We used BWA to align all Illumina sequencing reads to the assembled genome:

bwa mem -t 32 genome.fa R1.fq R2.fq | samtools sort -@ 32 -o aln.s.bam.

We used samtools view and fasta to extract read sequences from the alignments of the autosomes, chrX, and chrY.

for chr_num in $(seq 1 20); do

chr_name = "chr${ chr_num}"

samtools view -h aln.s.bam "${ chr_name }" |samtools fasta −1 "${chr_name}_R1.fasta'' −2 "${ chr_name}_R2.fasta".

cat *_R1.fasta > Autosome_R1.fa

cat *_R2.fasta > Autosome_R2.fa

samtools view -h -b aln.s.bam chrX | samtools fasta -1chrX_R1.fasta −2 chrX_R2.fasta

samtools view -h -b aln.s.bam chrY | samtools fasta -1chrY_R1.fasta −2 chrY_R2.fasta - Finally, we used meryl count on the collection of read data to build a k-mer database. The k value is chosen as 21.

meryl count k = 21 Autosome_R1.fa Autosome_R2.fa output Autosome.k21.meryl

meryl histogram Autosome.k21.meryl > Autosome.k21.hist.

We then generated CohortVCF:

$bwa mem -t 32 $ref_file $READ1 $READ2 | $samtools sort -n –threads $THREADS -o ${OUTPUT_PREFIX}.nsorted.bam -

$samtools fixmate -m ${OUTPUT_PREFIX}.nsorted.bam –threads $THREADS ${OUTPUT_PREFIX}.fixed.bam.

$samtools sort –threads $THREADS ${OUTPUT_PREFIX}.fixed.bam -o ${OUTPUT_PREFIX}.sorted.bam.

$samtools markdup –threads $THREADS ${OUTPUT_PREFIX}.sorted.bam ${OUTPUT_PREFIX}.marked.bam.

$picard AddOrReplaceReadGroups -I ${OUTPUT_PREFIX}.marked.bam -O ${OUTPUT_PREFIX}_gr.bam -RGID $OUTPUT_PREFIX -RGLB none -RGSM $OUTPUT_PREFIX -RGPL ILLUMINA -RGPU barcode.

$gatk LeftAlignIndels –input ${OUTPUT_PREFIX}_gr.bam –output ${OUTPUT_PREFIX}.realigned.bam –reference $ref_file

$gatk HaplotypeCaller –input ${OUTPUT_PREFIX}.realigned.bam –output ${OUTPUT_PREFIX}.g.vcf.gz –reference $ref_file –emit-ref-confidence GVCF

ls −1 *.g.vcf.gz |awk '{print "–variant",$1}' >arguments.file.

$gatk CombineGVCFs -R $ref_file –arguments_file arguments.file -o merged.g.vcf.gz.

$gatk GenotypeGVCFs -R $ref_file –variant merged.g.vcf.gz –output CohortVCF.vcf.gz.

$gatk HaplotypeCaller –input ${OUTPUT_PREFIX}.realigned.bam –output ${OUTPUT_PREFIX}.g.vcf.gz –reference $ref_file –emit-ref-confidence GVCF

ls −1 *.g.vcf.gz |awk '{print "–variant",$1}' >arguments.file.

$gatk CombineGVCFs -R $ref_file –arguments_file arguments.file -o merged.g.vcf.gz.

$gatk GenotypeGVCFs -R $ref_file –variant merged.g.vcf.gz –output CohortVCF.vcf.gz.

Gene prediction and annotation

The recent complete annotation of GRCr8 provided a source of annotation information for the SHRSP T2T assembly.[3] We utilized Liftoff (v1.6.3)[17] to carry over the annotations in the NCBI GRCr8 annotation (available at https://ftp.ncbi.nlm.nih.gov/genomes/all/GCF/036/323/735/GCF_036323735.1_GRCr8/GCF_036323735.1_GRCr8_genomic.gff.gz) using the -sc 0.97 flag to annotate duplicated gene copies of high similarity to the annotated gene.

We used BRAKER3 (v3.0.8) to predict protein-coding gene structures in our assemblies using long read RNA (Iso-Seq) to improve gene identification and protein evidence (OrthoDB v12, Vertebrata database) to train the gene prediction pipeline.[18]

Identification of unannotated novel genes

To identify potential novel genes in the T2T genomes, we extracted genes from each BRAKER annotation that exhibited no overlap with any gene from the Liftoff annotation using bcftools intersect -v. The output GFF3 file was then filtered to include only BRAKER gene entries that had at least 2 exons. We examine Iso-Seq RNA alignments to the genome assembly to identify potential coding sequences that were unannotated by Liftoff but were aligned with BRAKER annotations. Finally, we identified Iso-Seq RNA alignments to the assembly that were neither identified by Liftoff nor BRAKER3. In both cases, aligned Iso-Seq reads were in silico translated and similarity of the translated to known rat proteins was sought by NCBI Blastp.

Repetitive sequence annotation

Telomeric repeats were incorporated by the assembler. For further telomere analysis we adapted the Telomere Profiling method recently described.[22] To do so, telomere boundary coordinates were first identified and telomeric sequences were removed from the assembly using bedtools. The resulting telomere-trimmed chromosome ends, which consist of unique sequence, were then used as anchors to align Oxford Nanopore Technologies (ONT) reads with minimap2, enabling accurate assignment of reads to specific chromosome arms. Only primary and supplementary alignments were retained during read filtering (samtools view -F 256). We were then able to reliably extract ONT reads corresponding to all 44 chromosome arms. An ONT-specific algorithm was then applied to identify telomere boundary points directly on individual reads, enabling estimation of telomere length for each read. For each chromosome arm, mean and maximum telomere lengths were calculated using custom scripts, and the results are summarized in Table S2 (script file is available at Mendeley, see key resources table).

To identify known rodent repetitive elements withing the assembly we used RepeatMasker (v4.1.8). The genome was annotated with sequences in the Genetic Information Research Institute (GIRI) collection for Rattus and Rattus ancestral (1268 total repeat motifs). Regions of sequence self-similarity in the genome were identified using ModDotPlot software.[25] This tool is useful for identifying regions of the genome that have contain repetitive duplication such as centromeres. We could then compare the regions identified by ModDotPlot with regions annotated be RepeatMasker as being enriched in rat centromeric satellite repeat sequences. In addition, ModDotPlot identified several non-centromeric regions on autosome in which potential coding sequences have undergone repetitive duplication.

Identification of segmental duplications

We used Biser to analyze segmental duplications in the assemblies.[54] The resulting BEDPE file was processed to filter sequences greater than 1000bp and with a minimum identity of 75%. Effective duplication was as the total length of duplication multiplied by the percent identity of duplicated regions.

Scripts for identifying duplicated regions resistant to short-read evaluation

We generated a vcf file (CohortVCF.vcf) from alignment of short read datasets for 10 different rat strains (SHRSP, SHRB2, BN, F344, LE, LH, LN, SR-Jr, SS-Jr, WKY) to the SHRSP T2T assembly. This was used to run the following script at the unix command line:

gzip -cdf CohortVCF.vcf.gz | grep -v "#" | awk ‘NR = = 1{p = $2;q = $1; next} {print p,q, $2-p; p = $2; q = $1} END{print q,p}’ | awk ’{if($3 > 100000) print $0}’

This will identify records in the vcf file with greater than 100kb between variant calls. The script can be modified by changing the value of 100000 to whatever threshold that interests the researcher.

For each region identified, a subsequent script was used to count the reads for one of the source bam files within the region, and the number of reads within the region with mapping quality greater than 0. The results from this analysis are shown in Table S4.

samtools view <bamFile.bam> –min-MQ 1 <region of interest given by chrN:Start-End> | wc -l.

The above script will count reads that map with MQ > 1 to a region of interest. The bamFile.bam must be indexed. This too can be done by samtools.

samtools view <bamFile.bam> <region of interest given by chrN:Start-End> | wc -l.

This script will count all the reads mapped to the region of interest irrespective of the mapping quality. Variant deserts will have a signature where the read depth, as calculated by “TotalReadCount*ReadLength/(MappingEnd-MappingStart) would be near the read depth for the whole dataset, and “MQ > 0ReadCount*ReadLength/(MappingEnd-MappingStart) would be very near zero.

Pseudo-autosomal region analysis

We identified the chromosomal location of genes annotated on the human chr Y pseudoautosomal region (PAR1). Since none of these genes were located on rat chr Y we used ModDotPlot to compare sequence similarity in the proximal 10Mb of chrs X and Y. Such similarity is present in human X and Y, but was not found in the rat. RepeatMasker analysis of the distal part of chr Y indicated the accumulation of rat centromeric satellite repeats. Cytogenic techniques combined with fluorescent *in situ* hybridization were used to investigate alignment of rat chrs X and Y during meiosis.

Cytogenic visualization of centromeres and chromosome axes was used to investigate alignment of rat chrs X and Y during meiosis as described previously.[55] Briefly, testis tissue was dissected and the outer tunica punctured to release seminiferous tubules. Tubules were then incubated in a hypotonic solution (30 mm Tris, 50 mm sucrose, 17 mm citric acid, 5 mm EDTA, 2.5 mm dithiothreitol, 0.5 mm phenylmethanesulfonyl fluoride) for ∼45 min at room temperature). Tubules were then shredded in droplet of 100mM glucose to release single cells into solution. Cells were then fixed in a droplet of 1% paraformaldehyde/0.15% Triton X-100 (pH 9.2) and spread across the surface of cleaned glass slides. Slides were air-dried overnight in a humid chamber, rinsed briefly in 0.4% PhotoFlow (Kodak), and air-dried. Cells were then immunostained using the following primary antibodies diluted in 1x antibody dilution buffer [10x concentration: 2.5 mL normal donkey serum (Jackson ImmunoResearch, #017-000-121), 22.5mL 1x PBS, 0.75g bovine serum albumin (Fraction V; Fisher Scientific), and 12.5 μL Triton X-100]: 1:50 rabbit polyclonal antibody against MLH1 (Sigma-Aldrich PC56), 1:50 goat polyclonal antibody against SYCP3 (Santa Cruz Biotechnology # sc-20845), and 1:100 anti-human CREST (Antibodies Inc, Davis, CA #15–235). Secondary antibodies (Jackson ImmunoResearch, donkey anti-goat Rhodamine Red-X # 705-295-003, donkey anti-mouse FITC # 715-095-150, and donkey anti-human Coumarin AMCA # 709-095-149) were used at

1:200 dilution. Slides were washed three times in 1× PBS with constant agitation, rinsed briefly in distilled water, and mounted in several drops of ProLong Gold antifade media (Molecular Probes, Eugene, OR). Slides were then visualized on a Zeiss Axioskop microscope equipped with an AxioCam HRc camera and a 100× objective lens. See STAR Methods to access original image repository.

Pangenome graph construction and analysis

A rat pangenome was constructed using the Minigraph-Cactus pipeline (version 8.1.0b1).[39] Following the recommended workflow, chromosome-only sequences were first extracted from all assemblies and renamed accordingly, after which the required seqFile was generated. The SHRSP T2T assembly was designated as the reference/backbone genome. In addition to the default required parameters, the options –odgi –chrom-og clip full –vcf –gfa –gbz –giraffe –viz were specified, enabling the generation of the required.hal and.gbz files along with additional.vcf and.og files. Variants were subsequently processed using vcfbub (v0.1.2), which removes all non–top-level variant sites unless they occur within variants having a reference length greater than 10 kb.

Structural variant (SV) lengths and classifications were determined using bcftools in combination with custom scripts. For each variant record in the VCF, the reference allele (REF, column 4) length was first computed. The alternative allele(s) (ALT, column 5) were split by commas to obtain individual alleles, from which the minimum and maximum allele lengths were determined. Candidate insertion and deletion sizes were then calculated as follows: the insertion size was defined as the maximum between zero and (max_ALT_length − REF_length), and the deletion size was defined as the maximum between zero and (REF_length − min_ALT_length). The structural variant length (SVLEN) for the site was assigned according to the larger of these two values in absolute magnitude, with insertions given a positive SVLEN and deletions a negative SVLEN. Variants with an absolute SVLEN less than 50 bp or greater than 500,000 bp were excluded. The calculated SVLEN value was added to the INFO field of the VCF record, replacing any existing SVLEN entry to avoid duplication.

Structural variant analysis

Structural variant (SV) types were inferred from the AT tag in the VCF INFO column, which encodes the reference path (first entry) and one or more alternative paths (subsequent entries) as ordered node identifiers with orientation markers (">" for forward, "<" for reverse). For each variant record, node identifiers and orientations were extracted from each path. The classification proceeded in the following priority order: (i) duplication (DUP)—assigned if any alternative path contained repeated node identifiers (ignoring orientation); (ii) inversion (INV)—assigned if any alternative path contained at least one node in reverse orientation; (iii) deletion (DEL) or insertion (INS)—assigned by comparing the set and length of node identifiers in the first alternative path to those of the reference path: DEL was called if the alternative path's node set was a proper subset of the reference set and shorter in length; INS was called if the alternative path's node set was a proper superset of the reference set and longer in length. Variants not meeting any of these criteria, or lacking a valid AT field, were classified as complex (COMPLEX). Final visualizations were generated using R scripts. Notably, although duplications (DUP) were assigned the highest priority in the SV classification logic, only a single DUP event was identified (located at chr5:59,356,426). Due to its extremely small proportion relative to other SV types, it could not be displayed meaningfully in the R-based plots and was therefore excluded from the final figures.

We quantified genome-wide structural variant (SV) density and produced chromosome-level visualizations in R (v4.5.1) using vcfR, dplyr, data.table, and ggplot2. From the pangenome VCF, records were parsed with vcfR; positions (POS) were converted to numeric and SV lengths were extracted from INFO/SVLEN. Genotype fields were used to compute, per variant, the across-sample presence count (count_sv), defined as the number of samples without genotype "0" or "NA". SVs were then aggregated into non-overlapping 200 kb windows per chromosome (bin = floor(POS/2e5)), and the number of SVs per bin was calculated. The distribution of SV counts was summarized, with display capped at the 99th percentile to stabilize color scaling, and counts were rendered as filled rectangles spanning each 200 kb bin, colored along a viridis gradient. Gene features from the GFF were likewise binned in 200 kb windows by gene start coordinate. Counts were winsorized at the 95th percentile and linearly rescaled so that bar height corresponded to relative density, then drawn as orange bars above each chromosome track. Satellite repeat intervals from BED files were merged (gap $\leq$1 kb) and overlaid as magenta blocks on the gene-density track.

QUANTIFICATION AND STATISTICAL ANALYSIS

Statistical analyses were performed to assess relationships between genomic features. Correlations between structural variant (SV) density and gene density were evaluated using Spearman's rank correlation test with a bin size of 100 kb. Correlation coefficients (r) and associated *p*-values were calculated to assess statistical significance. Genome-wide and hotspot-restricted analyses were performed separately to compare patterns across different genomic contexts. Additional quantitative analyses, including SV density estimation, gene density calculations, and telomere length measurements, were performed as described in the method details. All statistical analyses were conducted using R (v4.5.1).

Cell Genomics, Volume *6*

Supplemental information

Telomere-to-telomere genome assembly

and a pangenome for the rat

Kai Li, Julia L. Ciosek, Sergey Koren, Adam M. Phillippy, Yaming Zhu, William A. Lauer, Shelise Y. Brooks, Gerard G. Bouffard, Brandon D. Pickett, Beth L. Dumont, Melissa L. Smith, Theodore S. Kalbfleisch, and Peter A. Doris

Supplemental Figure and Table legends

Fig. S1. An illustration of the various sequencing and bioinformatic processes used to create the assembly, **related to Star Methods –** Method Details: sequencing and assembly

Fig. S2. K-mer analysis by Meryl/Merqury to validate haploid genome, **related to Star Methods –** Method Details: sequencing and assembly

Fig. S3. ModDotPlot self-similarity analysis (color encoded with a calibrated spectrum included) to identify potential centromeric regions, **related to Star Methods –** Method Details: sequencing and assembly

Fig. S4. IGV session for a duplicated region on chr19 showing unshaded MQ=0 short reads that impede variant analysis and positionally accurate genotyping in this region, **related to Star Methods**: Scripts for identifying duplicated regions resistant to short read evaluation

Fig. S5. Non-allelic homologous recombination (NAHR) evaluation using NAHRwhals software in 4 genomic regions in which gene duplication events were observed, **related to Tables 3 and 4**, identification of novel genes

Table S1. Chromosome QV scores estimated by Merqury using the Illumina, HiFi and ONT reads from which the assembly was created, **related to Table 1B,** overall assembly statistics for the SHRSP T2T assembly compared to the prior SHRSP HiFi assembly

Table S2. Telomere lengths in the assembly, **related to Table 1B,** overall assembly statistics for the SHRSP T2T assembly compared to the prior SHRSP HiFi assembly

Table S3. Rat and mouse genome location of human PAR1 genes associated with chr X and Y, **related to Figure 3A,** PAR1 region of X and Y shows sequence similarity in humans, but not in rats

Table S4. Duplicated regions in the SHRSP T2T genome in which short read mapping results in extended regions of non-zero mapped reads that are >100kb, **related to Star Methods** : Scripts for identifying duplicated regions resistant to short read evaluation

Table S5. Duplicated regions are fewer in the previous assembly (GCA_021556685.1) constructed without ONT-UL data, **related to Star Methods** : Scripts for identifying duplicated regions resistant to short read evaluation

Fig. S1

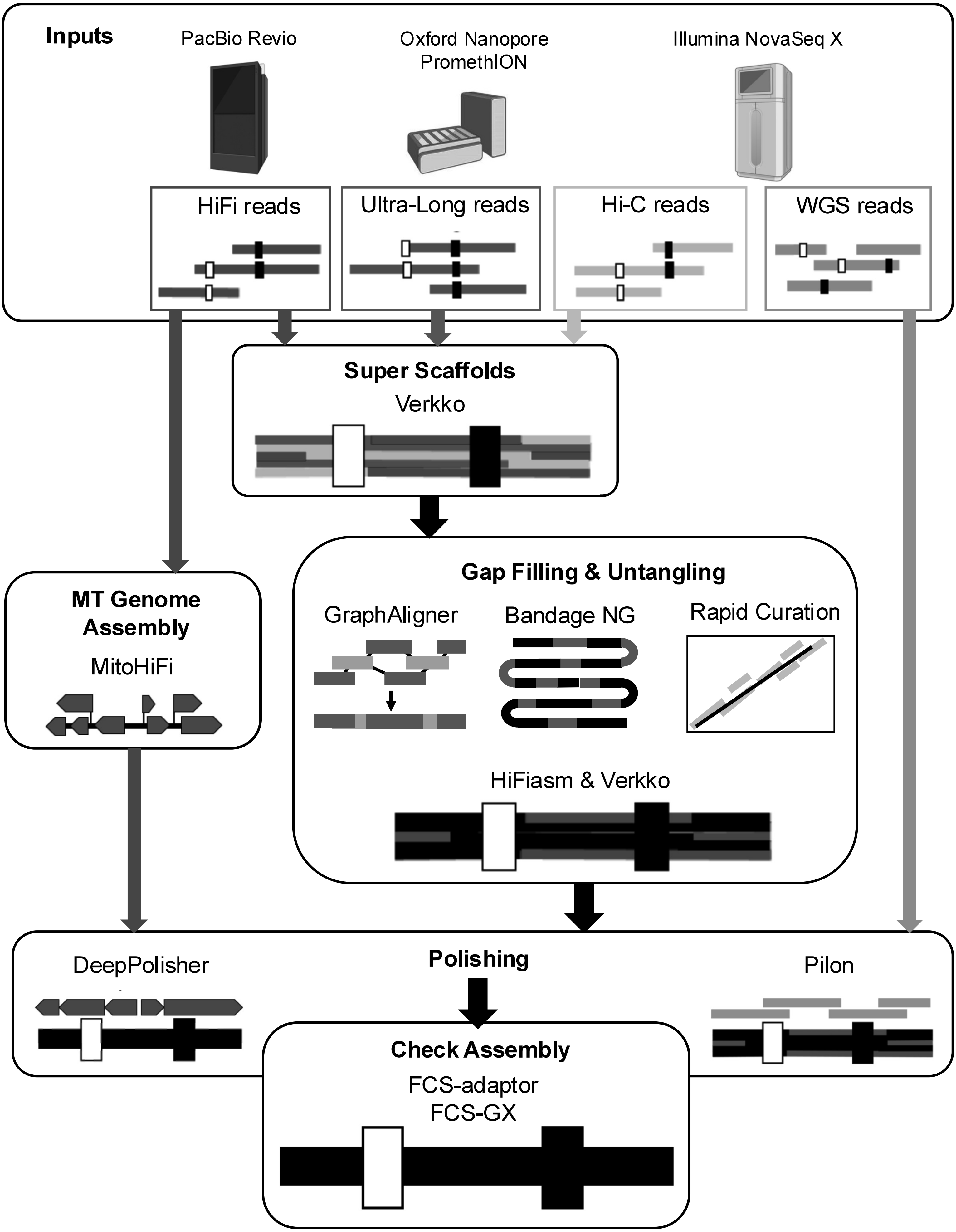

Inputs
PacBio Revio
Oxford Nanopore PromethION
Illumina NovaSeq X
HiFi reads
Ultra-Long reads
Hi-C reads
WGS reads
Super Scaffolds
Verkko
MT Genome Assembly
MitoHiFi
Gap Filling & Untangling
GraphAligner
Bandage NG
Rapid Curation
HiFiasm & Verkko
DeepPolisher
Polishing
Pilon
Check Assembly
FCS-adaptor
FCS-GX

Fig. S2 K−mer Frequency Distribution

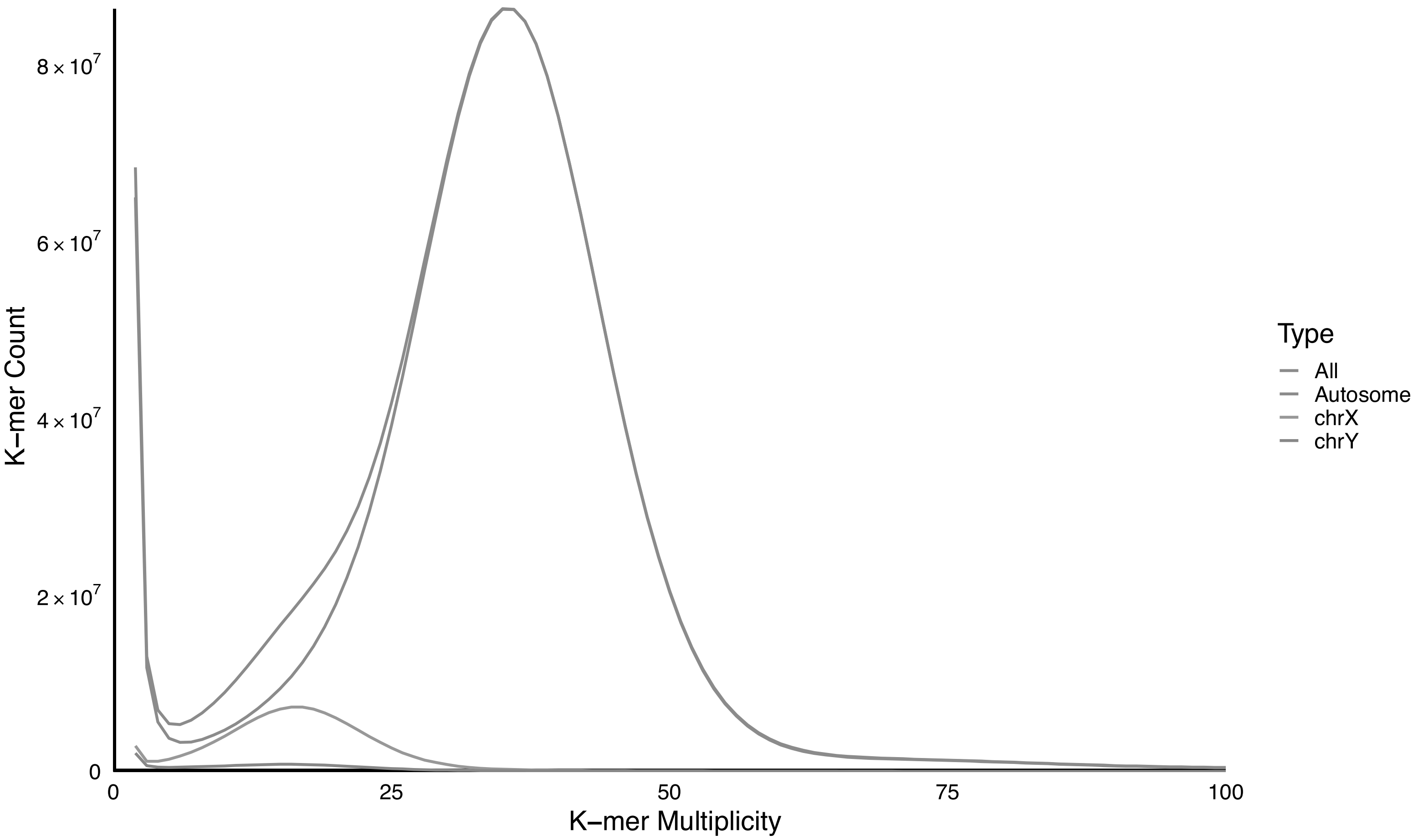

Fig. S3

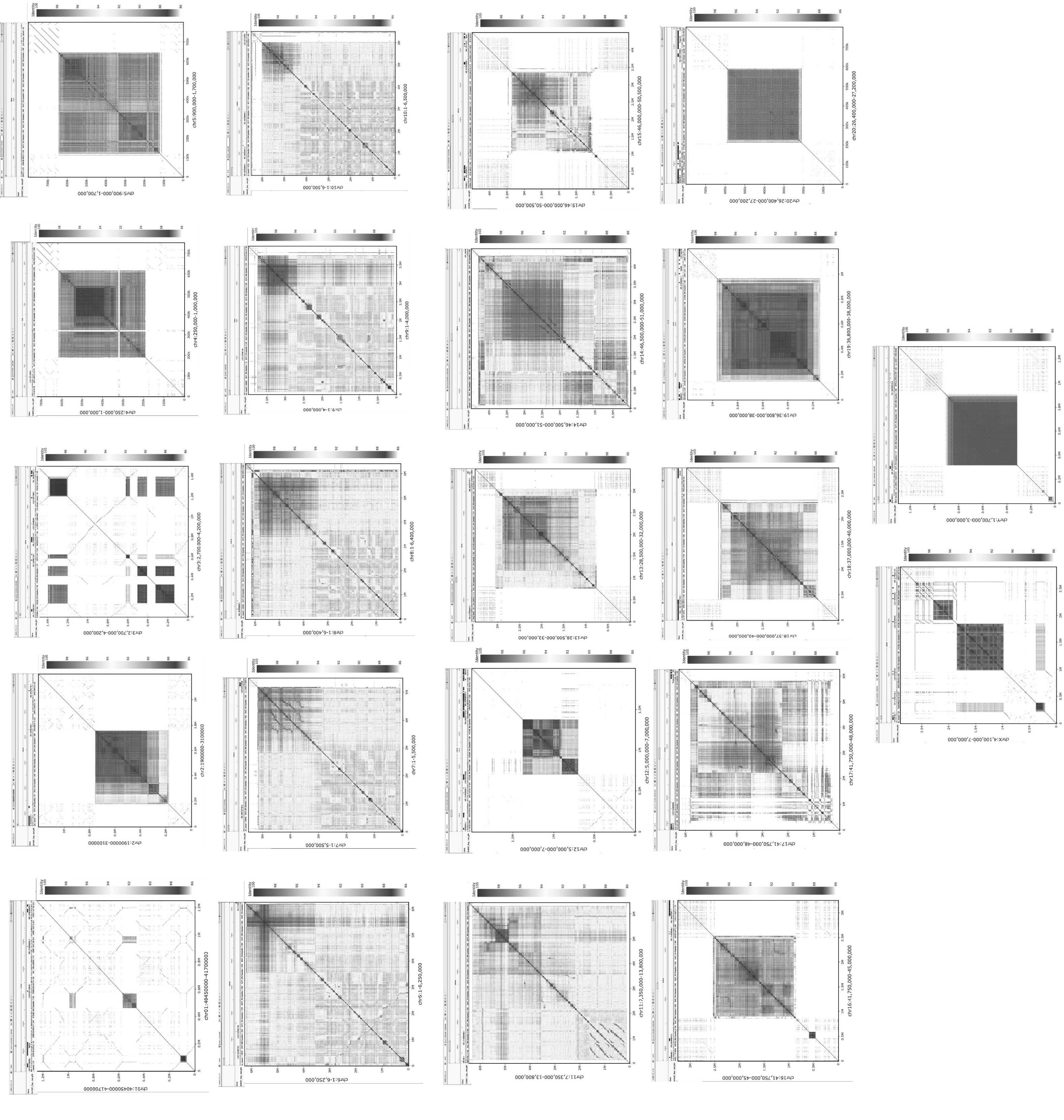

Fig. S4

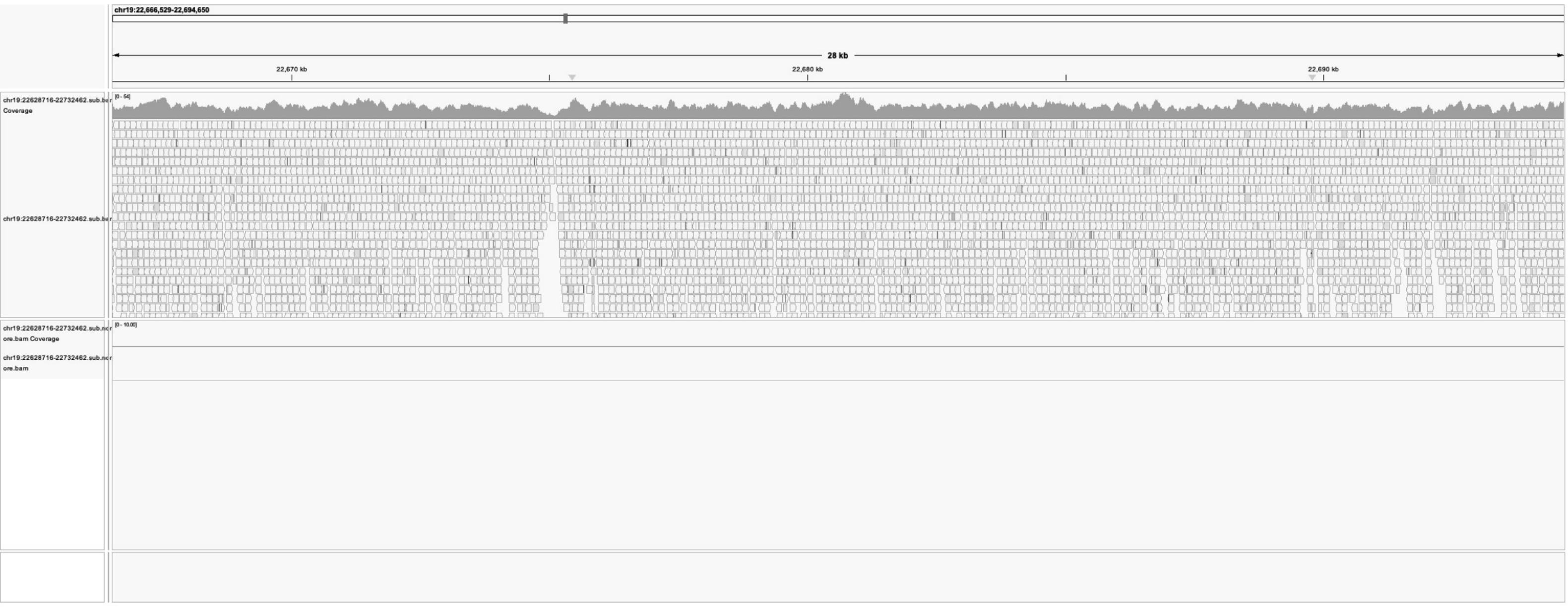

chr19:22,666,529-22,694,650
28 kb
22,670 kb
22,680 kb
22,690 kb
chr19:22628716-22732462.sub.ba
Coverage
[0 - 54]
chr19:22628716-22732462.sub.ba
chr19:22628716-22732462.sub.no
ore.bam Coverage
[0 - 10.00]
chr19:22628716-22732462.sub.no
ore.bam

Fig. S5

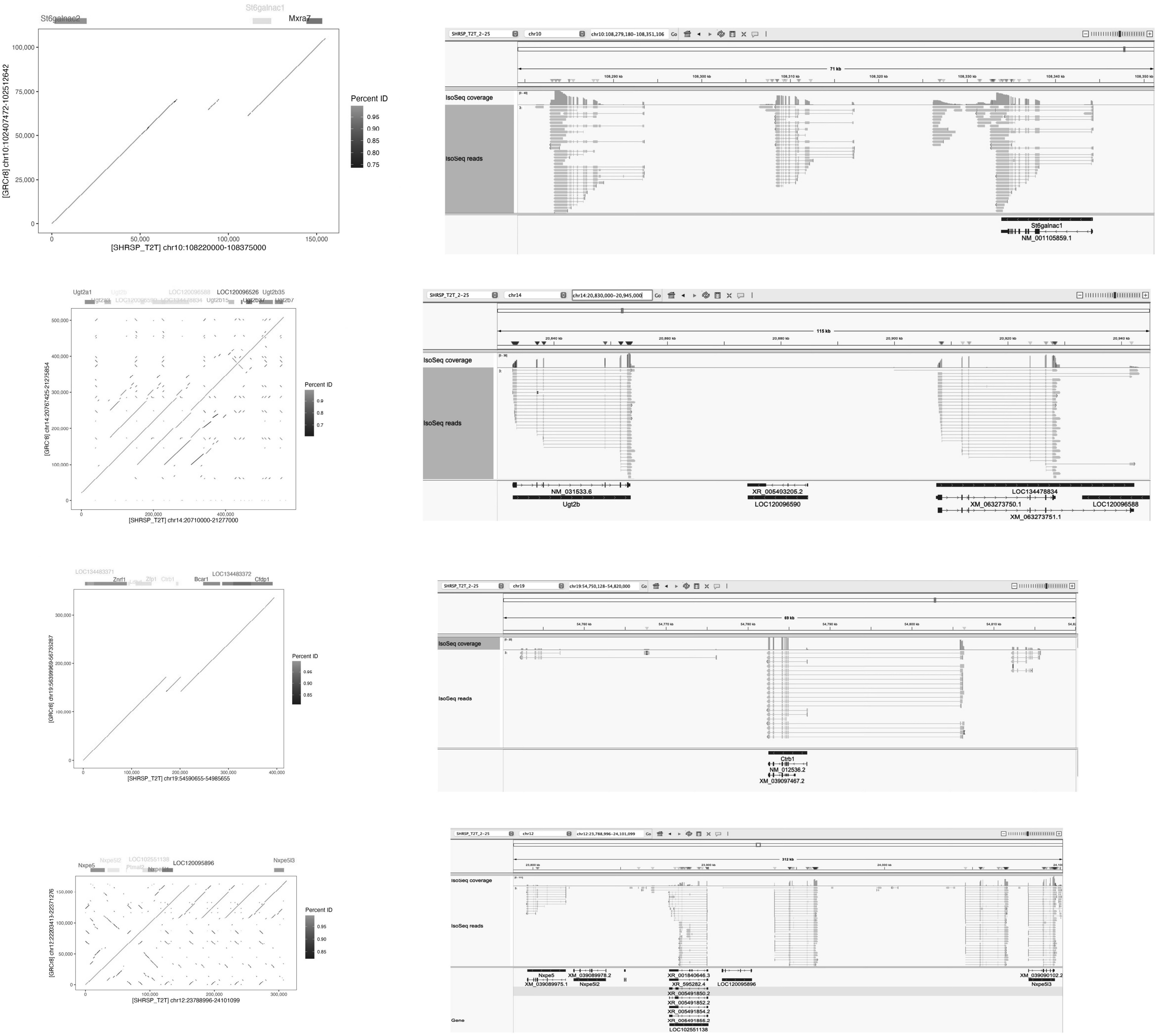

St6galnac2
St6galnac1
Mxra7
[GRCr8] chr10:102407472-102512642
[SHRSP_T2T] chr10:108220000-108375000
Percent ID
IsoSeq coverage
IsoSeq reads
St6galnac1
NM_001105859.1
Ugt2a1
LOC120096526
Ugt2b35
Ugt2b7
[GRCr8] chr14:20767425-21275854
[SHRSP_T2T] chr14:20710000-21277000
NM_031533.6
Ugt2b
XR_005493205.2
LOC120096590
LOC134478834
XM_063273750.1
XM_063273751.1
LOC120096588
Znrf1
Bcar1
LOC134483372
Cfdp1
[GRCr8] chr19:56399959-56733287
[SHRSP_T2T] chr19:54590655-54985655
Ctrb1
NM_012536.2
XM_039097467.2
Nxpe5
LOC120095896
Nxpe5l3
[GRCr8] chr12:22203413-22371276
[SHRSP_T2T] chr12:23788996-24101099
XM_039089975.1
XM_039089978.2
Nxpe5l2
XR_001840646.3
XR_595282.4
XR_005491850.2
XR_005491852.2
XR_005491854.2
XR_005491856.2
LOC102551138
XM_039090102.2
Gene

Table S1

QV of assembled genome

Chromosome	Uniq kmer	Total kmer	QV	Error rate
1	1225	273974597	66.7	2.13E-07
2	1191	251298673	66.5	2.26E-07
3	198	169841121	72.6	5.55E-08
4	487	184837503	69.0	1.25E-07
5	716	173011393	67.1	1.97E-07
6	133	146793962	73.7	4.31E-08
7	113	144522209	74.3	3.72E-08
8	107	131669335	74.1	3.87E-08
9	182	129549271	71.7	6.69E-08
10	84	113562547	74.5	3.52E-08
11	114	99913065	72.6	5.43E-08
12	185	53687143	67.8	1.64E-07
13	2901	109399675	59.0	1.26E-06
14	38	109260667	77.8	1.66E-08
15	33	107424533	78.3	1.46E-08
16	427	92830094	66.6	2.19E-07
17	65	94106387	74.8	3.29E-08
18	1245	85849053	61.6	6.91E-07
19	16	72640477	79.8	1.05E-08
20	271	57885805	66.5	2.23E-07
X	8184	162181918	56.2	2.40E-06
Y	1138	77330154	61.5	7.01E-07
Assembly QV=	65.0	Autosomes QV=	67.5	

Table S2

Telomeric repeat lengths by chromosome/arm in SHRSP T2T assembly. The average length was calculated using TeloBP (see Methods) and the maximum length is the length of the longest telomeric repeat read counted from the telomeric boundary identified by TeloBP.

	**Telomere length (bases)**			**Telomere length (bases)**	
	Average	Max		Average	Max
chr01_p-arm	31900	103106	chr01_q-arm	29651	74025
chr02_p-arm	26291	54939	chr02_q-arm	30758	76861
chr03_p-arm	10112	84021	chr03_q-arm	29189	63066
chr04_p-arm	29721	80928	chr04_q-arm	30582	72958
chr05_p-arm	38509	77090	chr05_q-arm	24833	147301
chr06_p-arm	34734	70787	chr06_q-arm	25271	72188
chr07_p-arm	37333	74893	chr07_q-arm	35225	70606
chr08_p-arm	28734	147301	chr08_q-arm	43705	128050
chr09_p-arm	33070	62410	chr09_q-arm	34782	147301
chr10_p-arm	37289	125297	chr10_q-arm	26701	80664
chr11_p-arm	32829	88879	chr11_q-arm	31954	78136
chr12_p-arm	33951	75358	chr12_q-arm	33576	83134
chr13_p-arm	37409	90113	chr13_q-arm	27669	74560
chr14_p-arm	32854	70048	chr14_q-arm	30669	63963
chr15_p-arm	31232	82048	chr15_q-arm	33368	85294
chr16_p-arm	48196	128050	chr16_q-arm	27686	68917
chr17_p-arm	36109	73528	chr17_q-arm	34513	103215
chr18_p-arm	30499	68127	chr18_q-arm	22984	51146
chr19_p-arm	41015	116710	chr19_q-arm	32817	77650
chr20_p-arm	30071	78195	chr20_q-arm	40097	123928
chrX_p-arm	39629	90148	chrX_q-arm	29676	62908
chrY_p-arm	33057	57004	chrY_q-arm	24481	65704

Table S3. Genomic location of genes associated with chr X and Y human PAR1 in rat and mouse genome assemblies. The first panel shows gene in the human PAR1 from the telomere to the region of Xg. The second panel shows other genes conserved in the adjacent human chrX and there location in mouse and rat assemblies.

Gene	Hsa RefSeq	GRCh38	Mmu RefSeq	GRCm39 position	Rn RefSeq	GRCr8 position	Rn RefSeq	SHRSP T2T position
PLCXD1	NM_018390	chrX:281381-303356	NM_207279.3	chr5:110247661-110253819	XM_006249594.4	chr12:52,238,611-52,241,876	XM_006249594.4	chr12:53,611,726-53,616,155
		chrY:281381-303356						
GTPBP6	NM_012227	chrX:304759-318796	NM_145147.5	chr5:110251843-11025606	NM_001135840.1	chr12:52,234,612-52,238,574	NM_001135840.1	chr12:53,607,932-53,612,854
		chrY:304759-318796						
PPP2R3B	NM_013239	chrX:333933-386907		absent	NM_001139492.1	chr14:103,483-110,740	NM_001139492.1	chr14:100,844-109,075
		chrY:333933-386907						
SHOX	NM_001191546	chrX:630898-644636	NM_001302357.	chr3:66879056-66889104	XM_001077477.5		XM_001077477.5	
		chrY:630898-644636				chrX:121,553,770-121,560,78		chrX:125,394,483-125,402,485
CRLF2	NM_022148	chrX:1190490-1212649	NM_001310694.	chr5:109702575-109706859	NM_134465.2	chr14:118,930-123,637	NM_134465.2	chr14:116,268-121,942
		chrY:1190490-1212649						
CSF2RA	NM_001161531	chrX:11268814-1305941	NM_009970.2	chr19:61212840-61216856	NM_001037660.1	chr14:113,073-118,247	NM_001037660.1	chr14:110,559-116,453
		chrY:1268814-1305941						
IL3RA	NM_002183	chrX:1336785-1382689	NM_008369.2	chr14:8114270-8123357	NM_139260.3	chr12:21,431,846-21,437,547	NM_139260.3	chr12:23,010,514-23,016,9
		chrY:1336785-1382689						
SLC25A6	NM_214418	chrX:1386596-1392042		absent	XM_039097301.1	chr18:70,846,358-70,847,597	XM_039097301.1	chr18:70,601,165-70,603,404
		chrY:1386157-1392042						
ASMTL	NM_004192	chrX:1403139-1452909		absent	NM_001105915.2	chr12:21,429,098-21,431,849	NM_001105915.2	chr12:23,007,850-23,011,513
		chrY:1403139-1452909						
P2RY8	NM_178129	chrX:1462581-1537185		absent		absent		absent
		chrY:1462581-1537185						
AKAP17A	NM_005088	chrX:1591604-1602520	NM_001418372.	chrX:169098201-169105547	NM_001127246.2	chr12:21,424,191-21,428,933	NM_001127246.2	chr12:23,002,870-23,007,915
		chrY:1591604-1602520						
ASMT	NM_001171038.2	chrX:1615059-1643081	NM_001308488.	chrX:169106356-169111844	NM_144759.2	chr12:21,418,488-21,423,338	NM_144759.2	chr12:22,997,036-23,003,004
		chrY:1615059-1643081						
DHRSX	NM_145177	chrX:2219506-2500976	NM_001033326.	chr4:156390377-156410432	XM_063271142.1	chr12:21,413,533-21,418,321	XM_063271142.1	chr12:22,992,105-22,997,868
		chrY:2219506-2500976						
ZBED1	NM_004729	chrX:2486435-2500976		absent		absent		absent
		chrY:2486435-2500976						
CD99	NM_002414	chrX:2691295-2741309	NM_138309.3	chrX:70463666-70536455	XM_063279501.1	chr20:56,000,484-56,005,135	XM_063279501.1	chr20:57,753,061-57,758,710
		chrY:2691295-2741309						
XG	NM_001141919	chrX:2752040-2816500		absent		absent		absent
		chrY:2752052-2752360						

Gene	Hsa RefSeq	GRCh38 Position	Mmu RefSeq	GRCm39 position	Rn RefSeq	GRCr8 position	Rn RefSeq	SHRSP T2T position
Gyg2	NM_001184704.2	chrX:2,828,430-2,883,318		absent		absent		absent
Arsd	NM_009589.5	chrX:2,903,472-2,929,839		absent		absent		absent
Arsl	NM_000047.3	chrX:2,934,309-2,968,745		absent		absent		absent
Arsh	NM_001011719.2	chrX:3,006,046-3,034,611		absent		absent		absent
Arsf	NM_001201538.2	chrX:3,039,734-3,113,227		absent		absent		absent
Mxra5	NM_015419.4	chrX:3,308,065-3,347,152		absent		absent		absent
Prkx	NM_005044.5	chrX:3,603,840-3,714,149	NM_001411694.1	chrX:76,804,078-76,840,045	NM_001033963.2	chrX:45,701,247-45,745,710	NM_001033963.2	chrX:49,612,943-49,657,417
Nlgn4	NM_001441322.1	chrX:5,889,542-6,229,367		absent		absent		absent
Vcx3a	NM_016379.4	chrX:6,533,118-6,535,618		absent		absent		absent
Pudp	XR_007068202.1	chrX:6,705,338-7,148,653	NM_026108.3	chr18:50,700,227-50,702,270	NM_001419508.1	chr18:46,064,367-46,067,747	NM_001419508.1	chr18:45,869,392-45,872,812
Sts	NM_001320752.2	chrX:7,146,790-7,355,141	NM_009293.1	chrX:168,855,832-169,015,537	NM_012661.1	chrX:46,102,024-46,111,368	NM_012661.1	chrX:50,028,597-50,037,940
Vcx	NM_013452.3	chrX:7,841,762-7,844,643		absent		absent		absent
Pnpla4	NM_004650.3	chrX:7,897,747-7,928,224		absent	NM_001401182.1	chrX:46,199,851-46,206,017	NM_001401182.1	chrX:50,131,310-50,137,481
Anos1	NM_001161531	chrX:8,528,374-8,732,637		absent	XM_002727786.4	chr10:69,058,951-69,060,798	XM_002727786.4	chr10:74,472,991-74,474,838
Fam9a	NM_174951.3	chrX:8,790,295-8,801,883		absent		absent		absent
Fam9b	NM_205849.3	chrX:9,023,732-9,034,627		absent	NM_001408910.1	chrX:138,541,821-138,555,147	NM_001408910.1	chrX:142,317,404-142,330,730
Tbl1x	NM_05647.4	chrX:9,462,795-9,720,240	NM_001426017.1	chrX:76,552,553-76,707,091	XM_039099666.2	chrX:45,452,483-45,610,032	XM_039099666.2	chrX:49,352,899-49,510,378
Gpr143	NM_001440781.1	chrX:9,724,846-9,779,102	NM_010951.3	chrX:151,564,417-151,592,142	NM_00116958.1	chrX:25,498,101-25,523,908	NM_00116958.1	chrX:31,429,925-31,922,719
Shroom2	NM_001649.4	chrX:9,785,929-9,949,943	NM_001290687.2	chrX:151,392,005-151,552,982	NM_001047893.3	chrX:25,307,663-25,479,924	NM_001047893.3	chrX:29,104,315-29,276,644
Wwc3	NM_0015691.5	chrX:10,014,754-10,144,974		absent	NM_001427611.1	chrX:27,145,518-27,263,946	NM_001427611.1	chrX:30,946,849-31,063,942
Clcn4	NM_001256994.2	chrX:10,156,475-10,238,160	NM_001357102.2	chr7:7,284,808-7,304,337	NM_001135840.1	chrX:27,290,269-27,357,467	NM_001135840.1	chrX:31,090,269-31,157,494
Mid1	NM_033290.4	chrX:10,444,810-10,834,183	NM_001425885.1	chrX:168,467,695-168,774,192	NM_022927.1	chrX:27,677,748-28,053,549	NM_022927.1	chrX:31,485,015-31,867,629

Table S4

Regions in this assembly that are effectively invisible for the purposes of variant analysis in genome wide association studies. These regions were identified by looking for distances >100kb between adjacent variants in a cohort VCF file called against the SHRSP T2T assembly using Illumina read data of 8 distinct inbred rat strains. The table lists regions in the SHRSP T2T genome that are >100kb and are invariant across strains.

Region	Total_Mapped Reads	Nonzero_Reads	Region_Length	Nonzero_Ratio	Depth_Include Zero_Reads
chr01:65528461-65828410	143,022	264	299,950	0.185%	46.7054
chr06:5375127-5547572	140,259	1137	172,446	0.811%	79.0851
chr08:0-101533	60,322	850	101,534	1.409%	21.4942
chr16:22944986-23138054	98,007	415	193,069	0.423%	49.6452
chr16:23152915-23333782	91,162	107	180,868	0.117%	49.3319
chr19:13788478-13988717	57,192	215	200,240	0.376%	27.914
chr19:13988718-14963881	246,526	27	975,164	0.011%	24.7821
chr19:14964518-15222277	64,371	2	257,760	0.003%	24.4403
chr19:15222278-15795262	145,384	65	572,985	0.045%	24.8215
chr19:15796756-16094218	77,606	2	297,463	0.003%	25.5164
chr19:16094789-16229615	34,119	0	134,827	0.000%	24.7506
chr19:16370485-16629041	66,845	2	258,557	0.003%	25.3348
chr19:16630401-17320124	164,482	37	689,724	0.022%	23.3737
chr19:17446618-18280396	202,076	4	833,779	0.002%	23.7504
chr19:18281443-19281592	247,717	42	1,000,150	0.017%	24.296
chr19:19282649-19916613	154,570	2	633,965	0.001%	23.9308
chr19:19916930-20408635	123,754	8	491,706	0.006%	24.6501
chr19:20599688-20708177	27,188	0	108,490	0.000%	24.4703
chr19:20708509-21649032	245,579	17	940,524	0.007%	25.5513
chr19:21904580-22083155	45,437	36	178,576	0.079%	24.9547
chr19:22083756-22341447	65,268	260	257,692	0.398%	24.7863
chr19:22356391-22592830	60,612	5	236,440	0.008%	25.1152
chr19:22628716-22732462	26,095	3	103,747	0.011%	24.6093
chr19:23005894-23193471	47,791	1	187,578	0.002%	24.9436
chr19:23511545-23671886	40,937	6	160,342	0.015%	25.0209

chr19:23672912-23798222	31,495	4	125,311	0.013%	24.5741
chr19:23801399-24277146	109,628	4	475,748	0.004%	22.6075
chr19:24409177-24530134	30,649	2	120,958	0.007%	24.8296
chr19:24604743-24711202	27,104	2	106,460	0.007%	24.9446
chr19:24770895-25326162	139,490	8	555,268	0.006%	24.583
chr19:25459227-25614553	39,514	6	155,327	0.015%	24.8812
chr19:25672011-25792543	34,003	26	120,533	0.076%	27.3368
chr19:25839357-25972814	33,038	6	133,458	0.018%	24.2523
chr19:25973876-26654632	173,897	140	680,757	0.081%	25.0414
chr19:26765190-27047607	96,337	10	282,418	0.010%	33.4786
chr19:27047639-27150332	36,398	116	102,694	0.319%	34.7554
chr19:27151213-27383024	78,759	0	231,812	0.000%	33.3559
chr19:27384256-27485273	33,380	7	101,018	0.021%	32.4317
chr19:27598238-27759195	57,443	0	160,958	0.000%	35.0311
chr19:27770887-27937813	58,514	3	166,927	0.005%	34.3925
chr20:39944072-40079376	70,996	392	135,305	0.552%	51.0319
chr20:40079812-40229066	77,718	528	149,255	0.679%	50.7071
chr20:57765266-57865713	105,542	8923	100,448	8.454%	33.098
chrX:0-197365	44,399	294	197,366	0.662%	15.574
chrX:198171-381930	16,859	50	183,760	0.297%	8.86722
chrX:383202-576381	18,286	38	193,180	0.208%	9.09314
chrX:586322-752104	23,877	56	165,783	0.235%	13.8111
chrX:795043-1121140	34,027	13	326,098	0.038%	10.0386
chrX:1413954-1617324	21,861	0	203,371	0.000%	10.3957
chrX:1617597-1726332	17,863	6	108,736	0.034%	15.7573
chrX:1727952-1885204	28,328	7	157,253	0.025%	17.5036
chrX:1971370-2179354	15,854	0	207,985	0.000%	7.38946
chrX:2192190-2395894	30,045	21	203,705	0.070%	14.1639
chrX:2545378-2733028	24,881	32	187,651	0.129%	12.7863
chrX:2733530-3149204	59,864	120	415,675	0.200%	13.9084
chrX:3149449-3267966	14,585	1	118,518	0.007%	11.8785
chrX:3268380-3744500	55,355	96	476,121	0.173%	11.1987

chrY:2016132-2127445	20,257	194	111,314	0.958%	17.9811
chrY:2267198-2514583	46,212	897	247,386	1.941%	18.4785
chrY:76844035-77331694	72,831	191	487,660	0.262%	11.363
		Sum of regions	17,363,793		

Table S5

Regions in this assembly >100kb between adjacent variants that are effectively invisible for the purposes of variant analysis in genome wide association studies are reduced when the cohort variants are called against the HiFi assembly of SHRSP (GCA_021556685.1). The table lists regions in the SHRSP HiFi genome that are invariant across strains. Significantly fewer invariant regions are called than in Table S4 reflecting the ability of the T2T assembly to more faithfully represent duplication events in the genome.

Region	Total Mapped Reads	Nonzero Reads	Region Length	Nonzero Ratio	Depth_Include Zero_Reads
chr1:1928585-2035493	21513	27	106909	0.126%	19.5536
chr1:2893349-3005595	24006	55	112247	0.229%	20.7701
chr2:3373804-3507174	39866	138	133371	0.346%	29.0775
chr3:182367925-182472286	36256	380	104362	1.048%	24.8097
chr3:182502840-182605066	20399	89	102227	0.436%	19.2104
chr4:186772362-186908652	24619	58	136291	0.236%	17.4876
chr4:186909387-187063658	22874	74	154272	0.324%	14.3632
chr6:142019143-142131637	45392	811	112495	1.787%	39.3922
chr7:54149-170508	23738	14	116360	0.059%	19.7701
chr7:229524-329888	21315	16	100365	0.075%	20.5728
chr7:2485313-2701255	615	57	215943	9.268%	0.264418
chr9:20230597-20341807	13083	433	111211	3.310%	11.4481
chr10:10667-167918	27656	106	157252	0.383%	16.9395
chr15:596915-706760	24471	18	109846	0.074%	21.5526
chr15:1105811-1222413	24712	54	116603	0.219%	20.465
chr15:7112818-7305166	4255	0	192349	0.000%	2.1544
chr16:960491-1082621	24687	55	122131	0.223%	19.5232
chr17:0-221720	39352	121	221721	0.307%	17.2219
chr17:376911-580750	40955	549	203840	1.340%	19.3812
chr19:14508370-14637801	10864	3	129432	0.028%	8.18922
chr20:56063334-56165450	19040	52	102117	0.273%	18.0339
chrY:2064322-2355205	54357	6864	290884	12.628%	17.6654
chrY:34604250-34776359	1456	0	172110	0.000%	0.820939
		Sum of regions	3324338		
